# Association Between Marijuana Use and Risk of Cancer: A Systematic Review and Meta-analysis

**DOI:** 10.1001/jamanetworkopen.2019.16318

**Published:** 2019-11-27

**Authors:** Mehrnaz Ghasemiesfe, Brooke Barrow, Samuel Leonard, Salomeh Keyhani, Deborah Korenstein

**Affiliations:** 1 Northern California Institute of Research and Education, San Francisco; 2 Currently a medical student at Warren Alpert Medical School of Brown University, Providence, Rhode Island; 3 Department of Medicine, University of California, San Francisco; 4 San Francisco Veterans Affairs Medical Center, San Francisco, California; 5 Department of Medicine, Memorial Sloan Kettering Cancer Center, New York, New York; 6 Weill Cornell Medical College, New York, New York

## Abstract

**Importance:**

Marijuana use is common and growing in the United States amid a trend toward legalization. Exposure to tobacco smoke is a well-described preventable cause of many cancers; the association of marijuana use with the development of cancer is not clear.

**Objective:**

To assess the association of marijuana use with cancer development.

**Data Sources:**

A search of PubMed, Embase, PsycINFO, MEDLINE, and the Cochrane Library was conducted on June 11, 2018, and updated on April 30, 2019. A systematic review and meta-analysis of studies published from January 1, 1973, to April 30, 2019, and references of included studies were performed, with data analyzed from January 2 through October 4, 2019.

**Study Selection:**

English-language studies involving adult marijuana users and reporting cancer development. The search strategy contained the following 2 concepts linked together with the AND operator: marijuana OR marihuana OR tetrahydrocannabinol OR cannabinoid OR cannabis; AND cancer OR malignancy OR carcinoma OR tumor OR neoplasm.

**Data Extraction and Synthesis:**

Two reviewers independently reviewed titles, abstracts, and full-text articles; 3 reviewers independently assessed study characteristics and graded evidence strength by consensus.

**Main Outcomes and Measures:**

Rates of cancer in marijuana users, with ever use defined as at least 1 joint-year exposure (equivalent to 1 joint per day for 1 year), compared with nonusers. Meta-analysis was conducted if there were at least 2 studies of the same design addressing the same cancer without high risk of bias when heterogeneity was low to moderate for the following 4 cancers: lung, head and neck squamous cell carcinoma, oral squamous cell carcinoma, and testicular germ cell tumor (TGCT), with comparisons expressed as odds ratios (ORs) with 95% CIs.

**Results:**

Twenty-five English-language studies (19 case-control, 5 cohort, and 1 cross-sectional) were included; few studies (n = 2) were at low risk of bias. In pooled analysis of case-control studies, ever use of marijuana was not associated with head and neck squamous cell carcinoma or oral cancer. In pooled analysis of 3 case-control studies, more than 10 years of marijuana use (joint-years not reported) was associated with TGCT (OR, 1.36; 95% CI, 1.03-1.81; *P* = .03; *I*^2^ = 0%) and nonseminoma TGCT (OR, 1.85; 95% CI, 1.10-3.11; *P* = .04; *I*^2^ = 0%). Evaluations of ever use generally found no association with cancers, but exposure levels were low and poorly defined. Findings for lung cancer were mixed, confounded by few marijuana-only smokers, poor exposure assessment, and inadequate adjustment; meta-analysis was not performed for several outcomes.

**Conclusions and Relevance:**

Low-strength evidence suggests that smoking marijuana is associated with developing TGCT; its association with other cancers and the consequences of higher levels of use are unclear. Long-term studies in marijuana-only smokers would improve understanding of marijuana’s association with lung, oral, and other cancers.

**Trial Registration:**

PROSPERO identifier: CRD42018102457

## Key Points

**Question:**

What is the association between marijuana use and cancer development in adults with at least 1 joint-year exposure (equivalent to 1 joint per day for 1 year)?

**Findings:**

This systematic review and meta-analysis identified 25 English-language studies assessing marijuana use and the risk for developing lung, head and neck, urogenital, and other cancers. In meta-analyses, regular marijuana use was associated with development of testicular germ cell tumors, although the strength of evidence was low; evidence regarding other cancers was insufficient.

**Meaning:**

Sustained marijuana use may increase the risk for testicular cancer, but overall, the association of marijuana use and cancer development remains unclear.

## Teaser

This systematic review and meta-analysis assesses the association of marijuana use with the development of cancer in adults with at least 1 joint-year exposure (equivalent to 1 joint per day for 1 year).

## Introduction

Marijuana is the most widely used illicit substance in the United States, with almost half of adults reporting lifetime use.^1^ Rates are increasing,^2^ with use among young adults (age range, 18-29 years) doubling from 10.5% in 2002 to 21.2% in 2014. Smoking remains the main route of marijuana exposure.^3,4^

Marijuana smoke and tobacco smoke share carcinogens, including toxic gases, reactive oxygen species, and polycyclic aromatic hydrocarbons, such as benzo[α]pyrene and phenols,^5^ which are 20 times higher in unfiltered marijuana than in cigarette smoke.^6^ The larger the puff volume, the greater the depth of inhalation,^7^ and longer breath-holding time with marijuana compared with cigarette smoking leads to higher tar and carbon monoxide exposure.^8^ Furthermore, marijuana use is associated with histopathologic bronchial inflammatory changes comparable to changes observed with smoking tobacco.^9^ Given that cancer is the second leading cause of death in the United States^10^ and smoking remains the largest preventable cause of cancer death (responsible for 28.6% of all cancer deaths in 2014),^11^ similar toxic effects of marijuana smoke and tobacco smoke may have important health implications.

Aside from shared properties with tobacco, marijuana use may alter cancer risk through other mechanisms. Tetrahydrocannabinol, the primary psychoactive ingredient in marijuana, may have adverse immunomodulatory effects^8,9^ associated with cancer. Two proto-oncogenes are overexpressed in the bronchial epithelium of marijuana-only smokers, with a higher frequency of gene expression compared with tobacco-only smokers.^8,12^ In contrast, cannabinoids, including tetrahydrocannabinol, can inhibit proliferation of some cancer cell types, impede angiogenesis in vitro, and reduce cancer growth in some animal models.^13,14^ The net association of marijuana use with developing cancer is unclear.

The increasing prevalence of marijuana use, particularly among young adults, raises concerns regarding whether using marijuana increases the risk for developing cancer. Despite increasing social acceptance of marijuana use, there remains a dearth of information on the association between marijuana consumption and health, including its association with incident cancer. We conducted a systematic review and meta-analysis to improve the understanding of the association of marijuana use with developing cancers.

## Methods

This systematic review and meta-analysis was consistent with the Preferred Reporting Items for Systematic Reviews and Meta-analyses (PRISMA) statement.^15^ The protocol was registered at PROSPERO at the start of our investigation.

### Data Sources and Searches

A systematic literature review was performed using studies found in a search of several online databases (PubMed, Embase, PsycINFO, MEDLINE, and the Cochrane Library), as well as references of the included studies. The search was conducted on June 11, 2018, and was updated on April 30, 2019. The studies were published from January 1, 1973, to June 11, 2018. We chose 1973 as the start date because Oregon decriminalized possession of marijuana in that year.^16^ For PubMed, Embase, MEDLINE, and the Cochrane Library, we used both controlled vocabulary and text words for synonymous terminology within titles and abstracts in the development of search strategies. In PsycINFO, we used text word searching of titles and abstracts. The search strategy contained the following 2 concepts linked together with the AND operator^17^: marijuana OR marihuana OR tetrahydrocannabinol OR cannabinoid OR cannabis; AND cancer OR malignancy OR carcinoma OR tumor OR neoplasm (eAppendix 1 in the Supplement). We combined search results using a bibliographic management tool (EndNote, version X9; Clarivate Analytics) and used the method by Bramer et al^18^ to eliminate duplicates.

### Study Selection

Two of us (M.G. and B.B.) independently screened all titles and abstracts for inclusion. We included studies published in English involving participants 18 years or older with at least 1 joint-year exposure (equivalent of 1 joint per day for 1 year) or more cumulative use (defined as ever use) of marijuana and reporting on the development of cancer. We excluded review articles, commentaries, case reports, case series, editorial articles, in vitro and animal studies, studies that did not primarily evaluate marijuana exposure or include information on cancer outcomes, studies that reported only outcomes after short-term exposure in a laboratory setting, and studies that included fewer than 10 marijuana users (eAppendix 5 in the Supplement). The same 2 reviewers (M.G. and B.B.) independently reviewed all full-text articles using predetermined inclusion and exclusion criteria. Additional articles were identified through author tracking of first and last authors and reference tracking. Disagreements regarding publication inclusion were resolved by discussion or referral to a third reviewer (D.K.) (eAppendix 2 in the Supplement).

### Data Extraction and Quality Assessment

For each included study, 2 of us (M.G. and B.B.) independently collected information on outcomes by cancer type (lung, head and neck, urogenital, and other cancers). They also extracted data on study design (eg, case-control vs cohort), study population, participant age, exposure route, marijuana use intensity and duration, percentage of marijuana-only smokers, confounders (eg, tobacco or alcohol use and occupational exposure), and funding source. Risk of bias (ROB) in individual studies was assessed independently by 3 of us (M.G., S.K., and D.K.) at both study and outcome levels using the Newcastle-Ottawa Scale for outcomes in observational studies.^19^ Disagreements were resolved by consensus. Studies were rated as having low ROB if they provided detail on exposure assignment (eg, marijuana-only smokers vs marijuana and tobacco smokers), had robust assessment and adjustment for key confounders, had sufficient follow-up for outcomes to occur, and quantified marijuana use in terms of joint-years of exposure (when presented) or years of use.

### Statistical Analysis

Data were analyzed from January 2 through October 4, 2019. The meta-analysis was performed if there were at least 2 studies of the same design (eg, case-control) addressing the same cancer without high ROB when heterogeneity was low to moderate for the following 4 specific cancers: lung cancer, head and neck squamous cell carcinoma (HNSCC), oral squamous cell carcinoma (SCC), and testicular germ cell tumor (TGCT). We extracted binary outcome odds ratios (ORs) or calculated them (with 95% CIs) when adequate data were provided. Narrative synthesis was performed when meta-analysis was not possible. We pooled data using a random-effects model. We used the method by Paule and Mandel^20^ to estimate τ^2^ and the method by Hartung and Knapp^21^ to adjust for small sample sizes. For meta-analyses with at least 2 studies, we performed the test for funnel plot asymmetry based on weighted linear regression using the efficient score and score variance described by Higgins et al^22^ and by Harbord et al.^23^ Statistical analysis was done using R statistical software (package “meta,” version 1.1.453; R Project for Statistical Computing). Heterogeneity was evaluated using forest plots and the *I*^2^ statistic; *I*^2^ values of 25%, 50%, and 75% were considered evidence of low, moderate, and high heterogeneity, respectively.^23^ Tests were 2-tailed and *P* < .05 was considered statistically significant. Three of us (M.G., S.K., and D.K.) discussed the overall strength of evidence for each outcome and graded it as insufficient, low, moderate, or high based on methods outlined by the Agency for Healthcare Research and Quality.^24^

## Results

### Literature Search

**Figure 1.**
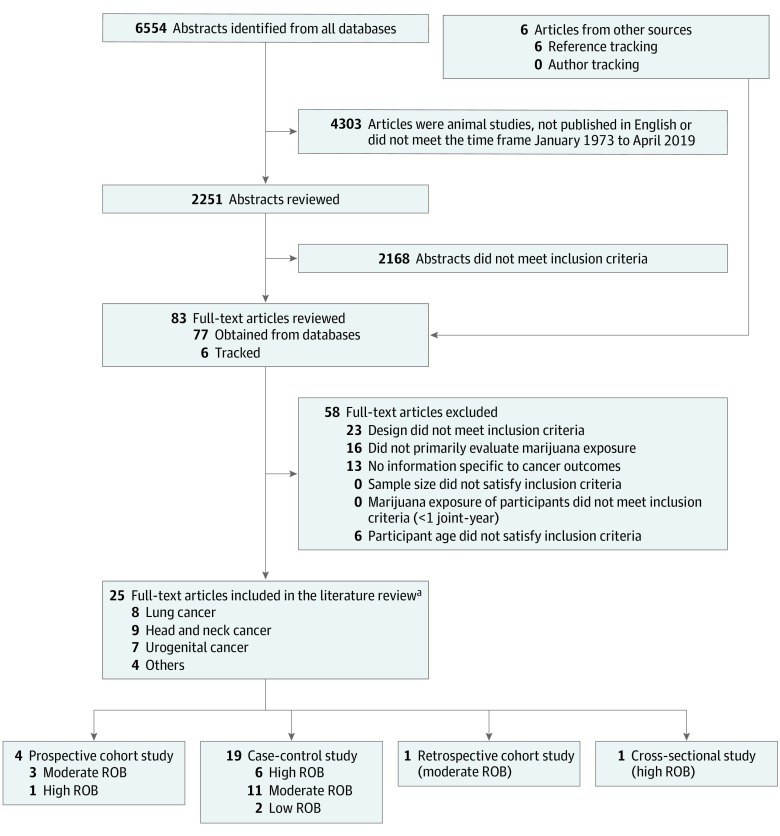
PRISMA Diagram of Evidence Search and Selection The flow of articles in the systematic review is shown. PRISMA indicates Preferred Reporting Items for Systematic Reviews and Meta-analyses; ROB, risk of bias. ^a^The number of full texts included in the literature review exceeds 25 because some studies were assigned to more than 1 outcome label and are counted twice.

Initial searches across databases identified 6554 abstracts; 25 studies ultimately met the inclusion criteria (Figure 1), including 19 case-control studies, 4 prospective cohort studies, 1 retrospective cohort study, and 1 cross-sectional study. Eight studies addressed risk of lung cancer, 9 addressed head and neck cancers, 7 addressed urogenital cancers, and 4 addressed other cancers (eAppendix 3 in the Supplement). All 25 included articles are described in eTable 1, eTable 2, eTable 3, eTable 4, eAppendix 4, eTable 5, and eTable 6 in the Supplement.

### Study Characteristics

Most studies were conducted in the United States (n = 16 [published 1993-2015]), followed by Europe (n = 3), northern Africa (n = 3), New Zealand (n = 2), and 1 from multiple countries. Methods of quantifying marijuana use varied (eg, frequency vs duration vs total joint-years). Two articles did not report the specific route of marijuana administration (eg, edible or smoked). Among those specifying exposure route (n = 23 [92%]), smoking predominated. We identified 19 distinct outcomes, of which 2 had sufficient supporting data from 2 or more studies and could be pooled in a meta-analysis. eAppendix 4, eTable 4 and eTable 5 in the Supplement describe ROB assessments for all included studies.

### Lung Cancer

Eight studies^25,26,27,28,29,30,31,32^ (1 prospective cohort, 1 retrospective cohort, 1 cross-sectional, and 5 case-control studies) examined the association between marijuana use and the development of lung cancer (Table 1 and eTable 1 in the Supplement). These studies were published between 1997 and 2015; the smallest was a case-control study with 33 lung cancer cases, and the largest was a prospective cohort study with 49 321 male participants. Three studies were undertaken in the United States, 2 in northern Africa, 1 in Sweden, 1 in New Zealand, and 1 in multiple countries. All studies had a moderate to high ROB and were generally limited by the small number of marijuana-only smokers (ie, most marijuana users also used tobacco), minimal exposure to marijuana, poorly described use assessment, and inadequate adjustment for confounders (Table 1). There were 405 individuals across case-control studies with more than 10 joint-years of marijuana use.

**Table 1.** Studies of Marijuana Use and Lung Cancer

Source	Population or Data Source	Study Design	Sample Size	Adjusted Risk for Lung Cancer With Marijuana Use	Risk of Bias	Comments
Callaghan et al,^25^ 2013	Swedish population based	Prospective cohort	49 321 Men	HR, 2.12 (95% CI, 1.08-4.14) with >50 lifetime episodes	High	1-Time use assessment, no results for marijuana-only smokers, 40-y follow-up period
Sidney et al,^26^ 1997	Kaiser Permanente, California	Retrospective cohort	64 855	RR, 0.9 (95% CI, 0.5-1.7) in men; RR, 1.1 (95% CI, 0.5-2.6) in women	Moderate	Minimal exposure, no results for marijuana-only smokers, short follow-up period of 8.6 y
Han et al,^32^ 2010	National US sample	Cross-sectional	29 195	OR, 7.87 (95% CI, 1.28-48.40) with ≥11 y of marijuana use	High	Unclear marijuana use assessment, no results for marijuana-only smokers, inadequate adjustment
Zhang et al,^29^ 2015	Multiple countries (United States, Canada, United Kingdom, and New Zealand)	Case-control	2159 Cases	OR, 0.54 (95% CI, 0.12-2.55) with ≥10 joint-years	High	Limited number of marijuana-only smokers (2 cases and 20 controls), inadequate adjustment
Aldington et al,^27^ 2008	New Zealand registry	Case-control	79 Cases	RR, 5.7 (95% CI, 1.5-21.6) with >10.5 joint-years	Moderate	Small sample of heavy users, no results for marijuana-only smokers
Hashibe et al,^28^ 2006	Los Angeles, California	Case-control	33 Cases	OR, 0.63 (95% CI, 0.32-1.2) with ≥60 joint-years	Moderate	Young participants, no results for marijuana-only smokers
Berthiller et al,^30^ 2008	Tunisia, Morocco, and Algeria	Case-control	430 Cases	OR, 2.3 (95% CI, 1.5-3.6)	High	Inadequate adjustment for confounders, unusual exposure form, no dose-response association seen
Voirin et al,^31^ 2006	Tunisia	Case-control	149 Cases	OR, 4.1 (95% CI, 1.9-9.0)	High	Inadequate adjustment for confounders, unusual exposure form, no dose-response association seen

Abbreviations: HR, hazard ratio; OR, odds ratio; RR, risk ratio.

Study results were mixed, and we were unable to pool data for this outcome. In general, studies were limited by low levels of marijuana exposure, little information about marijuana-only smokers, and other methodological flaws. Therefore, we concluded that evidence of the association between marijuana use and incident lung cancer was insufficient (Table 2).

**Table 2.** Strength of Evidence of Association of Marijuana Use and Each Type of Cancer

Outcome	Study Type	Evidence Strength	Comments
Lung cancer	1 Prospective study^29^ (high ROB), 1 retrospective observational cohort^26^ (moderate ROB), 5 case-control studies (2 moderate^27,28^ and 3 high^29,30,31^ ROB), and 1 cross-sectional study^32^ (high ROB)	Insufficient	1 Prospective study found an increased risk of lung cancer during a long period of follow-up; however, the study was limited by 1-time assessment of marijuana exposure and minimal exposure to marijuana. The retrospective study reported no association between marijuana use and an increased risk of lung cancer; however, the study was limited by minimal exposure to marijuana and the young age of participants. The case-control studies were limited by inadequate marijuana exposure, lack of information on the median marijuana exposure, limited results on marijuana-only smokers, and many other methodological flaws, with mixed findings. The cross-sectional study reported association between marijuana use and an increased risk of lung cancer; however, it was limited by unclear definitions in the marijuana assessment and no reported results on marijuana-only smokers.
HNSCC	4 Case-control studies (1 low^33^ and 3 moderate^34,35,36^ ROB)	Low	All studies rated as low or moderate ROB. Pooled data demonstrated that marijuana use exceeding 8 joint-years was associated with an increased risk of HNSCC.
Nasopharyngeal carcinoma	1 Case-control study^37^ (moderate ROB)	Insufficient	1 Case-control study demonstrated marijuana use was associated with increased risk of nasopharyngeal carcinoma; however, this study was limited by lack of reporting of the median marijuana exposure, inconsistent adjustment for important confounders, and potential bias in the selection of cases and controls
Oral cancer	4 Case-control studies (2 moderate^28,38^ and 2 high^39,40^ ROB)	Insufficient	Pooled data from moderate ROB studies demonstrated ever use of marijuana was not associated with an increased risk of oral cancer
Laryngeal cancer	1 Case-control study^28^ (moderate ROB)	Insufficient	1 Case-control study demonstrated marijuana use was not associated with increased risk of laryngeal cancer. However, results were not reported on marijuana-only smokers, and it was limited by a small sample of heavy marijuana users. The study did not report average marijuana exposure.
Pharyngeal cancer	1 Case-control study^28^ (moderate ROB)	Insufficient	1 Case-control study demonstrated marijuana use was not associated with increased risk of pharyngeal cancer. However, there were no results on marijuana-only smokers and no report of average marijuana exposure, and the study was limited by a small sample of heavy marijuana users.
Esophageal cancer	1 Case-control study^28^ (moderate ROB)	Insufficient	1 Case-control study demonstrated marijuana use was not associated with increased risk of esophageal cancer. However, results were not reported on marijuana-only smokers, the sample of heavy marijuana users was low, and average marijuana exposure was not reported.
Bladder cancer	1 Prospective cohort^41^ (moderate ROB)	Insufficient	1 Prospective study did not find association between marijuana use and increased risk of bladder cancer. The study was limited by inadequate adjustment for key confounders and 1-time assessment of marijuana exposure. The study did not report average marijuana exposure.
TGCT	3 Case-control studies^42,43,44^ (3 moderate ROB)	Low	Pooled data demonstrated more than a 10-y use of marijuana was associated with an increased risk of TGCT and nonseminoma TGCT.
Transitional cell carcinoma	1 Case-control study^45^ (low ROB)	Insufficient	1 Case-control study demonstrated marijuana use was associated with increased risk of transitional cell carcinoma. There were adequate marijuana exposure assessments and adjustment for confounders. The study was limited by few marijuana-only smokers.
Prostate cancer	1 Retrospective observational cohort^26^ (moderate ROB)	Insufficient	1 Retrospective study found association between marijuana use and increased risk of prostate cancer. The study was limited by lack of adjustment for key confounders, inadequate marijuana exposure, and no quantification of marijuana exposure.
Cervical cancer	1 Retrospective observational cohort^26^ (moderate ROB)	Insufficient	1 Retrospective study found association between marijuana use and increased risk of cervical cancer. The study was limited by lack of adjustment for key confounders, inadequate marijuana exposure, and no quantification of use.
Penile cancer	1 Case-control study^46^ (moderate ROB)	Insufficient	1 Case-control study did not demonstrate marijuana use was associated with increased risk of penile cancer. However, results were not reported on marijuana-only smokers, and it was limited by no quantification of use.
Kaposi sarcoma	1 Prospective cohort^47^ (moderate ROB)	Insufficient	1 Prospective study found association between weekly or more frequent use of marijuana and increased risk of Kaposi sarcoma. The study was limited by minimal marijuana exposure, young age of participants, and inadequate description of quantification of marijuana use.
Malignant primary adult-onset glioma	1 Prospective cohort^48^ (moderate ROB)	Insufficient	1 Prospective study found association between marijuana use and increased risk of malignant primary adult-onset glioma, but the study was limited by no quantification of marijuana use and no description of data collection.
Non-Hodgkin lymphoma	1 Case-control study^49^ (high ROB)	Insufficient	1 Case-control study did not demonstrate marijuana use was associated with increased risk of non-Hodgkin lymphoma. The study was limited by lack of information on dose and duration of use, inadequate marijuana exposure, and adjustment for key confounders.
Colorectal cancer	1 Retrospective observational cohort^26^ (moderate ROB)	Insufficient	1 Retrospective study did not find association between marijuana use and increased risk of colorectal cancer. The study was limited by lack of adjustment for key confounders, inadequate marijuana exposure, and no quantification of marijuana use.
Melanoma	1 Retrospective observational cohort^26^ (moderate ROB)	Insufficient	1 Retrospective study did not find association between marijuana use and increased risk of melanoma cancer. The study was limited by lack of adjustment for key confounders, inadequate marijuana exposure, and no quantification of marijuana use.
Breast cancer	1 Retrospective observational cohort^26^ (moderate ROB)	Insufficient	1 Retrospective study did not find association between marijuana use and increased risk of breast cancer. The study was limited by lack of adjustment for key confounders, inadequate marijuana exposure, and no quantification of marijuana use.

Abbreviations: HNSCC, head and neck squamous cell carcinoma; ROB, risk of bias; TGCT, testicular germ cell tumor.

### Head and Neck Cancer

**Figure 2.**
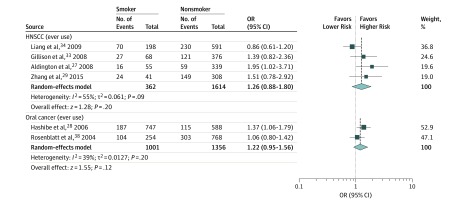
Association Between Marijuana Use and Risk of Developing Head and Neck Squamous Cell Carcinoma (HNSCC) and Oral Cancer in Case-Control Studies Included are 4 studies^27,29,33,34^ for HNSCC and 2 studies^28,38^ for oral cancer. The size of the boxes represents the weight of each study, and the diamond represents the overall effect. OR indicates odds ratio.

Nine case-control studies investigated the association of marijuana exposure with the development of head and neck cancers, including HNSCC, nasopharyngeal carcinoma, oral cancer, laryngeal cancer, pharyngeal cancer, and esophageal cancer; 1 of these studies evaluated multiple cancers^28^ (eTable 2 in the Supplement). Only 1 was rated as having a low ROB,^33^ and the number of cases ranged from 53 to 636. Four case-control studies (1 with low ROB^33^ and 3 with moderate ROB^34,35,36^) examined the association between marijuana use and HNSCC. All had sufficient supporting data for meta-analysis. Compared with nonsmokers, ever users of marijuana had similar risk of HNSCC (OR, 1.26; 95% CI, 0.88-1.80; *P* = .09; *I*^2^ = 55%) (Figure 2). The test for funnel plot asymmetry showed evidence of asymmetry (*P* = .045), with a bias coefficient of 3.48 (eFigure 1 in the Supplement). Findings among heavier users were mixed across studies (eTable 2 in the Supplement).

Four other case-control studies (2 with a moderate ROB^28,38^ and 2 with a high ROB^39,40^) evaluated marijuana exposure and risk of oral cancer. Pooled data from the 2 studies with moderate ROB^28,38^ revealed no association between ever use and oral cancer (OR, 1.22; 95% CI, 0.95-1.56; *P* = .12; *I*^2^ = 39%) (Figure 2); heterogeneity was moderate, and there was no evidence of funnel plot asymmetry (eFigure 1 in the Supplement). The 2 studies with a high ROB^39,40^ reported no association between marijuana use and the risk of oral SCC, but interpretability is limited by poor quantification of marijuana use and inadequate adjustment for confounders.

Nasopharyngeal carcinoma was examined in a 2004 case-control study with a moderate ROB^37^; a second population-based case-control study with a moderate ROB^28^ evaluated laryngeal, pharyngeal, and esophageal cancers. The study^37^ of nasopharyngeal carcinoma, which was performed in northern Africa and included 636 cases, found a higher risk of nasopharyngeal carcinoma with both ever marijuana consumption and lifetime high-dose marijuana smoking (≥2000 times; OR, 2.62; 95% CI, 1.00-6.86), after adjusting for tobacco and baseline variables. The study was limited by potential selection bias, inconsistent adjustment, and no reported results on marijuana-only smokers. The case-control study,^28^ which was based in Los Angeles, California, found no association of at least 30 joint-years of use with laryngeal, pharyngeal, or esophageal cancers, but it included too few such marijuana users (<10 users with ≥30 joint-years) to draw reliable conclusions (eTable 2 in the Supplement).

### Urogenital Cancer

**Figure 3.**
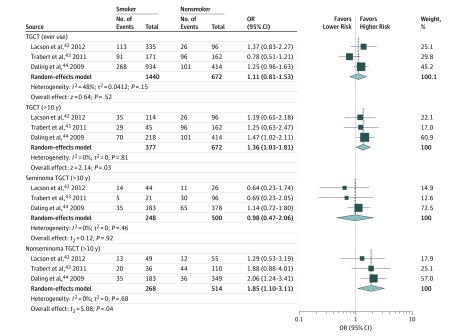
Association Between Marijuana Use and Risk of Developing Testicular Germ Cell Tumor (TGCT) in Case-Control Studies Included are 3 studies.^42,43,44^ The size of the boxes represents the weight of each study, and the diamond represents the overall effect. OR indicates odds ratio.

The association between marijuana use and developing urogenital cancer was evaluated in 1 prospective study,^41^ a retrospective study,^26^ and 5 case-control studies^42,43,44,45,46^ published between 1993 and 2015. Three case-control studies^42,43,44^ (with moderate ROB) assessed the association of marijuana use with TGCT; all of the studies included young participants and had a mean follow-up period of 6.6 years. In a pooled analysis (low heterogeneity), development of TGCT was not associated with ever use compared with never use (OR, 1.11; 95% CI, 0.81-1.53; *P* = .52; *I*^2^ = 48%), but it was associated with more than 10 years of marijuana use (OR, 1.36; 95% CI, 1.03-1.81; *P* = .03; *I*^2^ = 0%) (Figure 3). Subanalysis by histological type showed association of more than 10 years of marijuana use with the development of nonseminoma TGCT (OR, 1.85; 95% CI, 1.10-3.11; *P* = .04; *I*^2^ = 0%) but not seminoma TGCT (OR, 0.98; 95% CI, 0.47-2.06; *P* = .92; *I*^2^ = 0%) (Figure 3). There was no significant evidence of funnel plot asymmetry for TGCT (ever use) (*P* = .75), TGCT (>10 years) (*P* = .20), and seminoma TGCT (*P* = .09) (eFigure 2, eFigure 3, and eFigure 4 in the Supplement).

Other urogenital cancers were addressed in US-based single studies^26,41,45,46^ (eTable 3 in the Supplement). A prospective study^41^ (with moderate ROB) found that marijuana-only ever use was associated with a lower risk of bladder cancer (adjusted hazard ratio, 0.55; 95% CI, 0.31-1.00; *P* = .048), but the study was limited by inadequate adjustment for confounders. In a small study with low ROB,^45^ marijuana-only smoking was associated with transitional cell carcinoma (adjusted OR, 3.3), but there were only 10 marijuana-only smokers. Other studies with a moderate ROB found that marijuana use was associated with risk for prostate cancer (risk ratio [RR], 3.1; 95% CI, 1.0-9.5) and cervical cancer (RR, 1.4; 95% CI, 1.0-2.1)^26^ and was not associated with penile cancer,^46^ but study design issues limit reliability.

### Other Cancers

Four studies^26,47,48,49^ addressed marijuana use and the development of other cancers; all were performed in the United States (eTable 4 in the Supplement). A large, prospective study^48^ found an association between the development of malignant primary adult-onset glioma and weekly (n = 6002; RR, 3.2; 95% CI, 1.1-9.2) and monthly (n = 4699; RR, 3.6; 95% CI, 1.3-10.2) marijuana smoking compared with nonuse. Other studies found no association between marijuana ever use and breast cancer, colorectal cancer, and melanoma^26^ and non-Hodgkin lymphoma^49^; however, methodological concerns limit interpretation. Finally, a prospective study^47^ found that marijuana use among HIV-infected white men was associated with risk for developing Kaposi sarcoma (hazard ratio, 1.52; 95% CI, 0.99-2.32) in the 5-year lagged analysis. However, the study did not quantify exposure or report separately for marijuana-only smokers.

### Strength of Evidence

Low-strength evidence suggests that chronically smoking marijuana is associated with development of TGCT. Evidence on the association between marijuana use and other cancer types and evidence of the consequences of higher levels of use are insufficient (Table 2).

## Discussion

Although much is known about the association between tobacco smoke and cancer, less is known about the association between marijuana smoke and cancer. Both contain particulate matter and carcinogens. With increasing marijuana use and the high number of cancer-related deaths, understanding the association between marijuana use and cancer incidence is important. Low-strength evidence in the present systematic review and meta-analysis suggests that more than 10 years of marijuana use (joint-years were not reported) is associated with the development of TGCT. There was insufficient evidence to support an association between ever having used marijuana and other types of cancer. Available studies were limited by a small number of participants with high levels of use, poor use quantification, confounding related to cigarette smoking, and other methodological problems.

Our study contributes to the literature on the association between marijuana use and multiple cancers that has been examined individually in prior systematic reviews. Two systematic reviews^50,51^ examined the association between smoking marijuana and lung cancer. The first study^50^ offered evidence of biological plausibility (ie, molecular, cytomorphologic, and histopathologic changes); the second study^51^ noted pulmonary toxic effects and mixed evidence of an association with lung cancer but did not pool data to estimate an overall association. We found 1 meta-analysis^52^ examining the association of smoking marijuana with the development of head and neck cancer; it found no association but was limited by meta-analytic inconsistencies, pooling head and neck cancer subtypes in 1 plot and not addressing variable marijuana use, which may undermine its conclusions. Three other systematic reviews^53,54,55^ found an association between marijuana smoking and increased risk of TGCT but reported conflicting data on the association with other urogenital cancers. The present study confirms these findings and builds on the existing literature by assessing ROB, pooling data when feasible, and providing a clear picture of the gaps in evidence by rating the strength of the overall evidence.

Our systematic review and meta-analysis found insufficient evidence on the association between marijuana use and the development of lung cancer. There are biological reasons for concern about marijuana use and lung cancer. Several reports have documented changes in the bronchial epithelium of marijuana smokers that are similar to metaplastic premalignant alterations observed among tobacco smokers.^12,50,56^ Furthermore, histopathologic and molecular alterations and premalignant changes found in marijuana users,^12,57,58^ including mitotic figures, squamous cell metaplasia, and cell disorganization, suggest increased risk for respiratory neoplasm. In addition, marijuana joints with similar weight as tobacco cigarettes have higher tar burden, which may increase the carcinogenic risk.^8,59,60,61^ However, the difference in per weight tar burden is counterbalanced by the usual practice of smoking far fewer marijuana joints than tobacco cigarettes per day. Furthermore, lung cancer risk increases with both the number of daily cigarettes and the lifetime duration of smoking,^62^ with an increased risk only among those with high exposure. For example, a 40-year-old smoker of 1 pack per day (14 600 cigarettes) has a lung cancer risk approximately 20 times that of a nonsmoker. Our systematic review and meta-analysis included few marijuana smokers with similarly high exposure levels: there were 405 individuals across case-control lung cancer studies with more than 10 joint-years of use (3650 joints). Hence, low exposure burden, young participant age, and inadequate follow-up time in included studies may prevent detection of an association. Longitudinal cohorts with older populations of heavier marijuana users may be necessary to clarify the association of marijuana use with developing lung cancer.

Our findings are notable in a time of increasing marijuana use in the United States,^2,3,63^ with novel drug delivery methods, including vaping and edibles, becoming more popular, particularly in states that have legalized recreational use^4^ and among adolescents.^64,65^ However, most of the studies included in the present systematic review and meta-analysis are not recent, and smoking was the near-universal form of exposure. Vaped marijuana is believed to have fewer long-term toxic effects than smoked marijuana,^66^ but evidence is lacking. Although levels of tar are lower with marijuana use through vaping compared with smoking, vaporized marijuana can contain toxic levels of ammonia and heavy metals that may be associated with cancer, possibly cancers unrelated to smoking.^67,68,69^ Furthermore, with legalization may come heavier and more long-term use that may confer a higher risk for cancer. Misinformation may constitute an additional threat to public health; cannabis is being increasingly marketed as a potential cure for cancer in the absence of evidence,^70^ with enormous engagement in this misinformation on social media, particularly in states that have legalized recreational use.^71^ As marijuana smoking and other forms of marijuana use increase and evolve, it will be critical to develop a better understanding of the association of these different use behaviors with the development of cancers and other chronic conditions and to ensure accurate messaging to the public.

### Limitations

This systematic review and meta-analysis has limitations. Non–English-language articles were excluded; therefore, we may have overlooked relevant studies. Study populations were young, and few studies measured longitudinal exposure. The included studies were often limited by selection bias, recall bias, small sample of marijuana-only smokers, reporting of outcomes on marijuana users and tobacco users combined, and inadequate follow-up for the development of cancer. In addition, despite clear methodological differences across studies, we pooled some data. Although we used a conservative approach, these pooled estimates provide only a rough approximation of the association. Most studies poorly assessed exposure, and some studies did not report details on exposure, preventing meta-analysis for several outcomes. Understanding of the long-term health consequences of marijuana use could be improved by standardizing assessment tools to quantify use, including studies with larger samples of marijuana-only smokers, performing subanalysis based on form of use, and having longer follow-up times.

## Conclusions

Low-strength evidence suggests that smoking marijuana is associated with the development of TGCT; evidence of an association between marijuana use and incident lung cancer is of poor quality and inconclusive. Similarly, evidence regarding other cancer types is insufficient and is limited by low exposure and duration of follow-up. Increasing rates of marijuana use and evolution in delivery routes raise concerns about long-term consequences. Large-scale longitudinal studies with representative samples of marijuana-only smokers are needed to better understand the association of marijuana use with the development of lung, oral, and other cancers. In the meantime, clinicians should discuss marijuana use with patients to raise awareness of the lack of clarity on potential clinically important harms and to debunk beliefs in unproven benefits.

## Notes

Supplement. **eAppendix 1.** Search Strategies **eAppendix 2.** Study Selection **eAppendix 3.** Flow of Papers in the Review and Risk of Bias **eTable 1.** Studies That Examined Exposure to Marijuana and Development of Lung Cancer **eTable 2.** Studies That Examined Exposure to Marijuana and Development of Head and Neck Cancer **eTable 3.** Studies That Examined Exposure to Marijuana and Development of Urogenital Cancer **eTable 4.** Studies That Examined Exposure to Marijuana and Development of Other Cancers **eAppendix 4.** Quality Assessment Criteria and Risk of Bias Assessment **eTable 5.** Risk of Bias Assessment in Cohort and Cross-sectional Studies **eTable 6.** Risk of Bias Assessment in Case-Control Studies **eFigure 1.** Funnel Plot: Head and Neck Squamous Cell Cancer Case-Control Studies **eFigure 2.** Funnel Plot: Testicular Germ Cell Tumor Case-Control Studies **eFigure 3.** Funnel Plot: Testicular Germ Cell Tumor Case-Control Studies (>10 Years Use) **eFigure 4.** Funnel Plot: Testicular Germ Cell Tumor Case-Control Studies (Seminoma) **eReferences.** **eAppendix 5.** List of Excluded Studies
