# Error in Table 1

**DOI:** 10.1001/jamanetworkopen.2019.21065

**Published:** 2020-01-15

In the Original Investigation titled “Association Between Marijuana Use and Risk of Cancer: A Systematic Review and Meta-analysis,”^1^ published November 27, 2019, there was an error in the “comments” column of Table 1 regarding the study by Sidney et al. The comment should have appeared as “Minimal exposure, short follow-up period of 8.6 y.” This article has been corrected.^1^

## References

[1] Ghasemiesfe M, Barrow B, Leonard S, Keyhani S, Korenstein D Association between marijuana use and risk of cancer: a systematic review and meta-analysis. JAMA Netw Open. 2019;2(11):e1916318. doi:10.1001/jamanetworkopen.2019.16318 31774524 PMC6902836

