# Supplementary material for: Association Between Marijuana Use and Risk of Cancer: A Systematic Review and Meta-analysis
Source: JAMA Netw Open. 2019 Nov 27;2(11):e1916318. doi: 10.1001/jamanetworkopen.2019.16318 (PMC6902836; doi:10.1001/jamanetworkopen.2019.16318)
Supplement: Supplement. — eAppendix 1. Search Strategies eAppendix 2. Study Selection eAppendix 3. Flow of Papers in the Review and Risk of Bias eTable 1. Studies That Examined Exposure to Marijuana and Development of Lung Cancer eTable 2. Studies That Examined Exposure to Marijuana and Development of Head and Neck Cancer eTable 3. Studies That Examined Exposure to Marijuana and Development of Urogenital Cancer eTable 4. Studies That Examined Exposure to Marijuana and Development of Other Cancers eAppendix 4. Quality Assessment Criteria and Risk of Bias Assessment eTable 5. Risk of Bias Assessment in Cohort and Cross-sectional Studies eTable 6. Risk of Bias Assessment in Case-Control Studies eFigure 1. Funnel Plot: Head and Neck Squamous Cell Cancer Case-Control Studies eFigure 2. Funnel Plot: Testicular Germ Cell Tumor Case-Control Studies eFigure 3. Funnel Plot: Testicular Germ Cell Tumor Case-Control Studies (>10 Years Use) eFigure 4. Funnel Plot: Testicular Germ Cell Tumor Case-Control Studies (Seminoma) eReferences. eAppendix 5. List of Excluded Studies [file jamanetwopen-2-e1916318-s001.pdf]

Supplementary Online Content

Ghasemiesfe M, Barrow B, Leonard S, Keyhani S, Korenstein D. Association between marijuana use and risk of cancer: a systematic review and meta-analysis. *JAMA Netw Open*. 2019;2(11):e1916318. doi:10.1001/jamanetworkopen.2019.16318

eAppendix 1. Search Strategies
eAppendix 2. Study Selection
eAppendix 3. Flow of Papers in the Review and Risk of Bias
eTable 1. Studies That Examined Exposure to Marijuana and Development of Lung Cancer
eTable 2. Studies That Examined Exposure to Marijuana and Development of Head and Neck Cancer
eTable 3. Studies That Examined Exposure to Marijuana and Development of Urogenital Cancer
eTable 4. Studies That Examined Exposure to Marijuana and Development of Other Cancers
eAppendix 4. Quality Assessment Criteria and Risk of Bias Assessment
eTable 5. Risk of Bias Assessment in Cohort and Cross-sectional Studies
eTable 6. Risk of Bias Assessment in Case-Control Studies
eFigure 1. Funnel Plot: Head and Neck Squamous Cell Cancer Case-Control Studies
eFigure 2. Funnel Plot: Testicular Germ Cell Tumor Case-Control Studies
eFigure 3. Funnel Plot: Testicular Germ Cell Tumor Case-Control Studies (>10 Years Use)
eFigure 4. Funnel Plot: Testicular Germ Cell Tumor Case-Control Studies (Seminoma)
eReferences.
eAppendix 5. List of Excluded Studies

This supplementary material has been provided by the authors to give readers additional information about their work.

© 2019 Ghasemiesfe M et al. *JAMA Network Open*.

eAppendix 1. SEARCH STRATEGIES

DATABASES/WEBSITES:

PubMed
EMBASE
PsycINFO
MEDLINE
Cochrane Library

PubMed

Date Searched: Jun 11, 2018; Update: April 30, 2019

**Mesh terms**		
Marijuana OR Marihuana OR Tetrahydrocannabinol OR Cannabinoid OR Cannabis	AND	Cancer OR Malignancy OR Carcinoma OR Tumor OR Neoplasm

**#**	**Searches**	**Results**
#1	("cannabis"[MeSH Terms] OR "cannabis"[All Fields] OR "marijuana"[All Fields]) OR ("cannabis"[MeSH Terms] OR "cannabis"[All Fields] OR "marihuana"[All Fields]) OR ("dronabinol"[MeSH Terms] OR "dronabinol"[All Fields] OR "tetrahydrocannabinol"[All Fields]) OR ("cannabinoids"[MeSH Terms] OR "cannabinoids"[All Fields] OR "cannabinoid"[All Fields]) OR ("cannabis"[MeSH Terms] OR "cannabis"[All Fields])	47,585
#2	("neoplasms"[MeSH Terms] OR "neoplasms"[All Fields] OR "cancer"[All Fields]) OR ("neoplasms"[MeSH Terms] OR "neoplasms"[All Fields] OR "malignancy"[All Fields]) OR ("carcinoma"[MeSH Terms] OR "carcinoma"[All Fields]) OR ("tumour"[All Fields] OR "neoplasms"[MeSH Terms] OR "neoplasms"[All Fields] OR "tumor"[All Fields]) OR ("neoplasms"[MeSH Terms] OR "neoplasms"[All Fields] OR "neoplasm"[All Fields])	4,292,023
#3	(("cannabis"[MeSH Terms] OR "cannabis"[All Fields] OR "marijuana"[All Fields]) OR ("cannabis"[MeSH Terms] OR "cannabis"[All Fields] OR "marihuana"[All Fields]) OR ("dronabinol"[MeSH Terms] OR "dronabinol"[All Fields] OR "tetrahydrocannabinol"[All Fields]) OR ("cannabinoids"[MeSH Terms] OR "cannabinoids"[All Fields] OR "cannabinoid"[All Fields]) OR ("cannabis"[MeSH Terms] OR "cannabis"[All Fields])) AND (("neoplasms"[MeSH Terms] OR "neoplasms"[All Fields] OR "cancer"[All Fields]) OR ("neoplasms"[MeSH Terms] OR "neoplasms"[All Fields] OR "malignancy"[All Fields]) OR ("carcinoma"[MeSH Terms] OR "carcinoma"[All Fields]) OR ("tumour"[All Fields] OR "neoplasms"[MeSH Terms] OR "neoplasms"[All Fields] OR "tumor"[All Fields]) OR ("neoplasms"[MeSH Terms] OR "neoplasms"[All Fields] OR "neoplasm"[All Fields]))	2,907
#4	#1 AND #2	2,907
#5	#4 OR #3 AND ("humans"[MeSH Terms]	1,872

© 2019 Ghasemiesfe M et al. *JAMA Network Open*.

#6	#5 AND (("1973/01/01"[PDAT]: "2019/04/30"[PDAT])	1,869
#7	#6 AND English	1,788
#8	Search #7	

EMBASE

Date Searched: Jun 11, 2018; Update: April 30, 2019

#	Searches	Results
#1	'marijuana':ti,ab,kw OR 'marihuana':ti,ab,kw OR 'tetrahydrocannabinol':ti,ab,kw OR OR 'cannabis':ti,ab,kw 'cannabinoid':ti,ab,kw	53,092
#2	#1 AND [1973-2019]/py	53,568
#3	'cancer':ti,ab,kw OR 'malignancy':ti,ab,kw OR 'carcinoma':ti,ab,kw OR 'tumor':ti,ab,kw OR 'neoplasm':ti,ab,kw	3,475,110
#4	#3 AND [1973-2019]/py	3,454,746
#5	#1 AND #3	2,243
#6	#5 AND [1973-2019]/py	2,311
#7	#6 AND 'human'/de NOT 'nonhuman'/de	464
#8	('marijuana':ti,ab,kw OR 'marihuana':ti,ab,kw OR 'tetrahydrocannabinol':ti,ab,kw OR 'cannabinoid':ti,ab,kw OR 'cannabis':ti,ab,kw) AND ('cancer':ti,ab,kw OR 'malignancy':ti,ab,kw OR 'carcinoma':ti,ab,kw OR 'tumor':ti,ab,kw OR 'neoplasm':ti,ab,kw)	2,243
#9	#8 AND [1973-2019]/py	2,311
#10	limit 9 to human	465
#11	limit 10 to English	2
#12	limit 2 to human	4,481

PsycINFO

Date Searched: Jun 11, 2018; Update: April 30, 2019

#	Searches	Results
1	ab (Marijuana OR Marihuana OR Tetrahydrocannabinol OR Cannabinoid OR Cannabis)	24,174
2	1 AND (("1973/01/01"[PDAT]: "2019/04/30"[PDAT])	23,561
3	ab (Cancer OR Malignancy OR Carcinoma OR Tumor OR Neoplasm)	98,654
4	3 AND (("1973/01/01"[PDAT]: "2019/04/30"[PDAT])	96,803
5	1 AND 3	599
5	ab (Marijuana OR Marihuana OR Tetrahydrocannabinol OR Cannabinoid OR Cannabis) AND (Cancer OR Malignancy OR Carcinoma OR Tumor OR Neoplasm) AND (("1973/01/01"[PDAT]: "2019/04/30"[PDAT])	461
6	1 AND 3 AND Limit to human	461

© 2019 Ghasemiesfe M et al. *JAMA Network Open*.

7	(Marijuana OR Marihuana OR Tetrahydrocannabinol OR Cannabinoid OR Cannabis) AND (Cancer OR Malignancy OR Carcinoma OR Tumor OR Neoplasm) AND human AND (("1973/01/01"[PDAT]: "2019/04/30"[PDAT])	461
8	7 AND Limit to English	461

MEDLINE

Date Searched: Jun 11, 2018; Update: April 30, 2019

#	Searches	Results
1	(("cannabis"[MeSH Terms] OR "cannabis"[All Fields] OR "marijuana"[All Fields]) OR ("cannabis"[MeSH Terms] OR "cannabis"[All Fields] OR "marihuana"[All Fields]) OR ("dronabinol"[MeSH Terms] OR "dronabinol"[All Fields] OR "tetrahydrocannabinol"[All Fields]) OR ("cannabinoids"[MeSH Terms] OR "cannabinoids"[All Fields] OR "cannabinoid"[All Fields]) OR ("cannabis"[MeSH Terms] OR "cannabis"[All Fields])) AND (("neoplasms"[MeSH Terms] OR "neoplasms"[All Fields] OR "cancer"[All Fields]) OR ("neoplasms"[MeSH Terms] OR "neoplasms"[All Fields] OR "malignancy"[All Fields]) OR ("carcinoma"[MeSH Terms] OR "carcinoma"[All Fields]) OR ("tumour"[All Fields] OR "neoplasms"[MeSH Terms] OR "neoplasms"[All Fields] OR "tumor"[All Fields]) OR ("neoplasms"[MeSH Terms] OR "neoplasms"[All Fields] OR "neoplasm"[All Fields])) AND medline[sb]	2,397
2	1 AND (("1973/01/01"[PDAT]: "2019/04/30"[PDAT])	2,390
3	Limit 2 to human	1,866
4	Limit 3 to English	1,788

Cochrane Library

Date Searched: Jun 11, 2018; Update: April 30, 2019

#	Searches	Result
1	*Marijuana OR Marihuana OR Tetrahydrocannabinol OR Cannabinoid OR Cannabis AND Cancer OR Malignancy OR Carcinoma OR Tumor OR Neoplasm*	805
2	1 AND [Jan 1973- April 2019]	805
3	2 AND human	805
4	4 AND Cochrane reviews (Protocols only), Trials, Methods Studies, Technology assessments, Economic Evaluations and Cochrane Groups AND NOT Cochrane reviews (Reviews)	50
5	4 AND English	0

© 2019 Ghasemiesfe M et al. *JAMA Network Open*.

eAppendix 2. STUDY SELECTION

Inclusion and Exclusion criteria and process

1. Is the article published in English?

   No -> STOP. Excluded (Excluded study language)
   Yes-> Proceed to 2.

2. Does the intervention or exposure consist of cannabis variants including plant-based marijuana, marihuana in any form (smoking, vapor, edible, or extract) or tetrahydrocannabinol (THC) extract?

   No -> STOP. Excluded (Not relevant to topic)
   Yes -> Proceed to 3.

3. Is the article about “synthetic” cannabis, THC or marijuana?

   Yes -> STOP. Excluded (Not relevant to topic)
   No -> Proceed to 4.

4. Is the article of any following study designs or publication types?

   - Case report
   - Case series study
   - Review article
   - Opinion/Editorial
   - In-vitro and animal study

   No -> Proceed to 5.
   Yes-> STOP. Excluded (Excluded study design or publication type)

5. Are most the study subjects younger than age 18?

   No -> Proceed to 6.
   Yes -> STOP. Excluded

6. Does cumulative exposure to marijuana greater than or equal to 1 joint-year?

   No -> STOP. Excluded
   Yes -> Proceed to 7.

7. Do studies report outcomes follow acute exposure in a laboratory setting?

   Yes -> STOP. Excluded
   No -> Proceed to 8.

8. Do studies contain sample size less than ten subject?

© 2019 Ghasemiesfe M et al. *JAMA Network Open*.

Yes -> STOP. Excluded
No -> Proceed to 9.

9. Does the study report any of the following outcomes? The list below includes outcomes of interest:

9-1 Smoking Related Cancers:
- Lung cancer – Bronchogenic carcinoma, Non-Small-Cell lung carcinoma, Small Cell lung carcinoma, Multiple Pulmonary Nodules, Pancoast Syndrome, Pulmonary Sclerosing Hemangioma, Pleural neoplasms, Malignant Pleural Effusion

-Colorectal cancer – Colonic neoplasms, Sigmoid neoplasms, Hereditary Nonpolyposis colorectal neoplasms, Rectal neoplasms, Adenomatous Polyposis Coli

-Urogenital cancer – Urinary bladder neoplasms, Kidney neoplasms, Ureteral neoplasms, Urethral neoplasms, Penile neoplasms, Prostatic neoplasms, Testicular neoplasms, Fallopian Tube neoplasms, Ovarian Neoplasms, Uterine neoplasms, Vaginal neoplasms, Vulvar neoplasms

-Head and Neck cancer – Esophageal neoplasms, Facial neoplasms, Mouth neoplasms, Tracheal neoplasms, Thyroid neoplasms, Otorhinolaryngologic neoplasms

9-2 Other Common Cancers

-Breast carcinoma in situ, Breast Ductal carcinoma, Breast Lobular carcinoma, Hereditary Breast and Ovarian Cancer Syndrome, Inflammatory Breast neoplasms, Unilateral Breast neoplasms, Triple Negative Breast neoplasms, Prostate neoplasms

9-3 Other/ All cancers

-Soft Tissue Neoplasms, Skin Neoplasms, Nervous System Neoplasms, Hematologic Neoplasms, Endocrine Gland Neoplasms, Digestive System Neoplasms, Bone Neoplasms, Intestinal Neoplasms, Abdominal Neoplasms

No -> STOP. Excluded
Yes -> Proceed to 10.

10. Does the study design a randomized clinical trial, clinical trial, experimental study, case-control, prospective cohort study, retrospective cohort study, cross-sectional, cross-sectional cohort or case crossover study?

No -> STOP. Excluded
Yes -> STOP. Included

© 2019 Ghasemiesfe M et al. *JAMA Network Open*.

eAppendix 3. Flow of papers in the review and risk of bias

Figure 1: Flow diagram of outcomes and risk of bias identified in the review

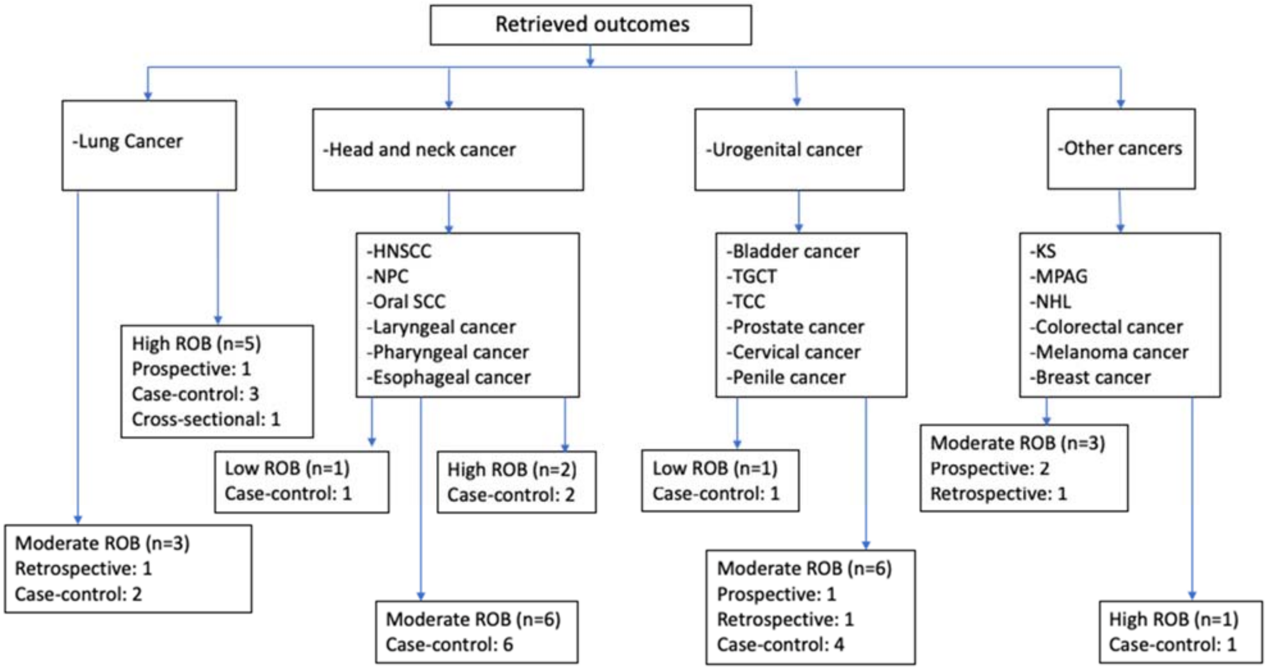

- HNSCC-Head and Neck Squamous Cell Carcinoma, NPC-Nasopharyngeal Cancer, Oral SCC-Oral Squamous Cell Cancer, TGCT-Testicular Germ Cell Tumor, TCC-Transitional Cell Carcinoma, KS-Kaposi's Sarcoma, NHL-Non-Hodgkin's Lymphoma, MPAG-Malignant Primary Adult-onset Glioma, ROB-Risk of Bias
- Two papers had multiple outcomes.

© 2019 Ghasemiesfe M et al. *JAMA Network Open*.

eTable 1. Studies that examined exposure to marijuana and development of Lung cancer

Study Year Design	Study Population	Sample Size, n	Age (years)	Average MJ exposure/ % of MJ only users	Confounders and baseline variables	Adjustment	Follow-up	Study Results	Risk of Bias	Funding source
Zhang et al, 2015, Case-control[29]	Primary incidental lung cancer and controls	2,159 cases, 2,985 controls	Mean: 55.2± 10.5	Not specified/ 17.1 % (370/2159) cases, 45.5% (1358/2985) controls	1.Age<br>2.Gender<br>3.Race<br>4.Education<br>5.Tobacco<br>6.Alcohol	Inadequate	N/A	• Smoking MJ in MJ-only smokers was not associated with all types of lung cancer (OR=1.03, 95% CI 0.51–2.08) after adjusting for baseline variables.<br>• Smoking MJ in MJ-only smokers was not associated with all types of lung cancer among > 1 joint/day smokers (OR=0.49, 95% CI 0.11–2.25) and > 20 years smokers (OR=1.64, 95% CI 0.45–6.00) after adjusting for baseline variables.	High	NIH, CCSRI, USPHS, ARF, SECMC and WPHCC, SBLF, SMSKCC
Callaghan et al, 2013, Prospective[25]	Swedish volunteer from Patient Register, NCDR, and the Total Population Register	49,321	18-20 at start of study	Not specified/ 1.4 % (689/49321)	1.SES<br>2.Tobacco<br>3.Alcohol<br>4.Respiratory conditions	Adequate	40 years	• Smoking MJ was associated with increased risk of lung cancer over a 40-year follow-up period in heavy (> 50 times) users (HR=2.12, 95 % CI 1.08–4.14) after adjusting for tobacco and baseline differences.	High	OMH, CAMH, SCWLSR
Han et al, 2010, Cross-sectional[32]	NSDUH participants from 2005-2007	29,195	35-49	Not specified/ Not specified	1.Age<br>2.Gender<br>3.Race<br>4.Education<br>5.SES<br>6.Tobacco<br>7.Alcohol<br>8.Durations of non-medical use of pain killers, tranquilizers, stimulants, and sedatives	Adequate	N/A	• Smoking MJ and duration of MJ use were associated with lung cancer after adjusting for key confounders and baseline variables.<br>• Smoking MJ for ≥11 years was associated with increased risk of lung cancer in people aged 35 to 49 compared with non-MJ smokers (AOR=7.87, 95% CI 1.28–48.40) after adjusting for key confounders and baseline variables.<br>• Smoking MJ for 2-10 years was not associated with increased risk of lung cancer in people aged 35 to 49 compared with non-MJ smokers (AOR=2.12, 95% CI 0.41–10.95) after adjusting for key confounders and baseline variables.	High	None
Aldington et al, 2008, Case-control[27]	Lung cancer cases and cancer-free controls	79 cases, 324 controls	Range 35-55	Not specified/ Not specified	1.Age<br>2.Gender<br>3.Race<br>4.Tobacco<br>5.FH of lung cancer	Adequate	5 years	• Smoking MJ was not associated with increased risk of lung cancer among lifetime users (> 20 joints) (RR=1.2, 95% CI 0.5–2.6) after adjusting for tobacco and other baseline variables.<br>• Smoking MJ was associated with increased risk of lung cancer in highest tertile of	Moderate	NMH, HBMRF

© 2019 Ghasemiesfe M et al. *JAMA Network Open*.

								exposure (> 10.5 joint-years) (RR=5.7, 95% CI 1.5–21.6) after adjusting for tobacco and other baseline variables.<br>• Smoking MJ was associated with 8% increased risk of lung cancer with each joint-year of use (RR=1.08, 95% CI 1.02–1.15) after adjusting for tobacco and other baseline variables.		
Berthiller et al, 2008, Case-control[30]	Lung cancer cases and non-cancer hospitalized controls	430 cases, 755 controls	Mean: 59.4± 11 Morocco, 57± 11.6 Tunisia, 64.4± 11.3 Algeria	5 joints/month Tunisia, 9 joints/month Algeria/ 0%	1.Age<br>2.Gender<br>3.Tobacco<br>4.Occupational exposure<br>5.Place of residence	Inadequate	N/A	• Smoking MJ was associated with increased risk of lung cancer (OR=2.4, 95% CI 1.6–3.8) after adjusting for tobacco and baseline variables.<br>• The association persisted (OR=2.3, 95% CI 1.5–3.6) even after adjustment for lifetime tobacco use.	High	None
Hashibe et al, 2006, Case-control[28]	Lung cancer cases and cancer-free controls	1,212 cases, 1,040 controls	Majority 45-≤54 years, range: 18-65	Not specified/ Not specified	1.Age<br>2.Gender<br>3.Race<br>4.Education<br>5.Tobacco<br>6.Alcohol	Adequate	N/A	• Smoking 50 joint-years of MJ was not associated with all types of lung cancer (AOR=1.0, 95% CI 0.74-1.4) after adjusting for tobacco and baseline variables.<br>• Smoking MJ was not associated with all types of lung cancer (AOR=0.62, 95% CI 0.32-1.2) in individuals who smoked > 60 joint-years after adjusting for tobacco and baseline variables.	Moderate	NIH, ARF
Voiron et al, 2006, Case-control[31]	Lung cancer cases and non-cancer hospitalized controls	149 cases, 188 controls	Mean: 57±12	Not specified/ Not specified	1.Age<br>2.Tobacco<br>3.Occupational exposure	Inadequate	N/A	• Past MJ smoking was associated with lung cancer (OR=4.1, 95% CI 1.9 –9.0) after adjusting for tobacco and baseline variables.	High	None
Sidney et al, 1997, Retrospective [26] ‡	Participants from KPMCP-NC	64,855	Mean: 33	40.1% (10,710/26,733)/ Not specified	1.Age<br>2.Race<br>3.Education<br>4.Tobacco<br>5.Alcohol	Adequate	Mean: 8.6 years	• Ever use of smoked MJ was not associated with increased risk of lung cancer [(men: RR=0.9, 95% CI 0.5-1.7), (women: RR=1.1, 95% CI 0.5-2.6)] compared to non-MJ and non-tobacco smokers after adjusting for key confounders and baseline variables.	Moderate	NIDA, NCI, ABMRF

Steps for Breath, the Labrecque Foundation (SBLF), The Society of Memorial Sloan-Kettering Cancer Center (SMSKCC), The Ontario Ministry of Health (OMH), The Swedish Council for Working Life and Social-Canadian Cancer Society Research Institute (CCSRI), Alper Research funds (ARF), Sheffield Experimental Cancer Medicine Centre (SECMC), Weston Park Hospital Cancer Charity (WPHCC), Steps for Research (SCWLSR), Long-Term Care to the Centre for Addiction and Mental Health (CAMH), The New Zealand Ministry of Health (NMH), The Hawke's Bay Medical Research Foundation (HBMRF), National Cause-of-Death Register (NCDR), National Cancer Institute (NCI), The National Institute on Drug Abuse (NIDA), The Alcoholic Beverage Medical Research Foundation (ABMRF), Socioeconomic Status (SES), Kaiser Permanente Medical Care Program of Northern California (KPMCP-NC), National Institutes of Health (NIH), National Surveys on Drug Use and Health (NSDUH), Marijuana (MJ), Family History (FH)

*All comparisons are to never users unless specified otherwise

†All studies used structured questionnaire to assess MJ exposure

‡Not specified route of exposure

© 2019 Ghasemiesfe M et al. *JAMA Network Open*.

eTable 2. Studies that examined exposure to marijuana and development of head and neck cancer

Study Year Design	Study Population	Sample Size	Age (years)	Average MJ exposure/ % of MJ only users	Confounders and baseline variables	Adjustment	Outcome Examined	Follow-up	Study Results	Risk of Bias	Funding source
Liang 2009[34] Case-control †	HNSCC cases and controls	434 Cases, 547 controls	Mean: 60.3 ± 11.42	Not specified/ Not specified	1.Age 2.Gender 3.Race 4.Education 5.Tobacco 6.Alcohol 7.FH of cancer 8.HPV16 serology	Adequate	HNSCC	4 years (1999-2003)	• Current MJ smoking was associated with lower risk of HNSCC (OR=0.52, 95% CI 0.34-0.80, p≤0.001) after adjusting for key confounders and baseline variables. • Smoking MJ (10 to 20 years) was associated with lower risk of HNSCC (OR=0.38, 95% CI, 0.22-0.67) after adjusting for key confounders and baseline variables. • Smoking MJ (moderate weekly use) was associated with lowerd risk of HNSCC ($OR_{0.5\text{-}<1.5 \text{ times vs. } <0.5 \text{ time}}$ =0.52, 95% CI=0.32–0.85) after adjusting for key confounders and baseline variables.	Moderate	NIH, FAMRI
Feng 2009[40] Case-control	NPC cases and cancer-free controls	636 Cases, 615 controls	N/A	Not specified/ Not specified	1.Age 2.Gender 3.SES 4.Tobacco 5.Dietary factors	Adequate	NPC	3 years (2001–2004)	• Ever use of smoked MJ was associated with increased risk of NPC (p=0.025) after adjusting for tobacco and baseline variables. • High-dose lifetime MJ smoking (≥ 2000 times) was associated with higher NPC risk (OR=2.62, 95% CI 1.00 –6.86) after adjusting for tobacco and baseline variables.	Moderate	ICR
Gillison 2008[33] Case-control †	HNSCC cases and cancer-free controls	240 Cases, 322 controls	50-65	Not specified/ Not specified	1.Age 2.Gender 3.Race 4.Tobacco 5.Alcohol 6.Tooth loss 7.Frequency of tooth brushing 8.# of oral sex partners	Adequate	HNSCC	6 years (2000-2006)	• Smoking MJ among MJ-only smokers was associated with increased risk of HNSCC in HPV-16 positive patients (OR=4.5, 95% CI 1.6 -13) after adjusting for key confounders and baseline variables. • In MJ smokers, increasing intensity (joints per month, P trend = 0.007) and duration (in years, P trend = 0.011) of MJ use	Low	DRCRFCI, SMCRF, NIDCR

© 2019 Ghasemiesfe M et al. *JAMA Network Open*.

									were associated with increased risk of HNSCC in HPV-16 positive patients. • Smoking MJ among MJ-only smokers (≥ 5 joint-years) was associated with 11-fold increased risk of HNSCC in HPV-16 positive patients (OR=11.0, 95% CI 1.6-74) compared with sporadic users or non-users after adjusting for key confounders and baseline variables.		
Aldington 2008[35] Case-control †	HNSCC cases and cancer-free controls	75 cases, 319 controls	Range: ≤39–55	Median: 25 years among cases, 10.5 years among controls/ Not specified	1.Age 2.Gender 3.Race 4.SES 5.Tobacco 6.Alcohol	Adequate	HNSCC	4 years (2001-2005)	• Smoking MJ in highest tertile of exposure (>8.3 joint-years) was not associated with increased risk of HNSCC (RR=1.6, 95% CI, 0.5 to 5.2) after adjusting for key confounders and baseline variables. • Smoking MJ was not associated with increased risk of HNSCC among individuals who used MJ 5 years before diagnosis (RR=1.08, 95% CI 0.77-1.53) after adjusting for key confounders and baseline variables.	Moderate	NMH, HBMRF,GlaxoSmithKline (UK)
Hashibe 2006[28], Case-control †	Upper aerodigestive tract cancer cases and cancer-free controls	1,212 cases, 1,040 controls	Majority 45 to ≤54 years, range: 18-65	Not specified/ Not specified	1.Age 2.Gender 3.Race 4.Education 5.Tobacco 6.Alcohol	Adequate	Oral SCC Laryngeal cancer Pharyngeal cancer Esophageal cancer	N/A	• Smoking MJ was not associated with oral cancer (AOR=1.1, 95% CI 0.56-2.1) or laryngeal cancer (AOR=0.84, 95% CI 0.28 - 2.5) in individuals who smoked > 60 joint-years after adjusting for key confounders and baseline variables. • Smoking MJ was not associated with pharyngeal cancer (AOR=0.57, 95% CI 0.20 - 1.6) and esophageal cancer (AOR=0.53, 95% CI 0.22-1.3) in individuals who smoked > 30 joint-years after adjusting for key confounders and baseline variables.	Moderate	NIH, ARPEG of the UCLA JCCC
Llewellyn 2004[39],	Oral SCC cases and cancer-	116 Cases,	Mean: 38.8± 5.7	Not specified/Not	1.Age 2.Gender	Inadequate	Oral SCC	7 years (1990 and	• Smoking MJ was not associated with increased risk of oral SCC	High	NHSE-LRO, R

© 2019 Ghasemiesfe M et al. *JAMA Network Open*.

Case-control (AA)	free controls	207 controls		specified	3.Tobacco<br>4.Alcohol			1997)	(OR=1.0, 95% CI 0.5—2.2) after adjusting for tobacco and alcohol consumption.		and D, and RFP
Llewellyn 2004[38] Case-control (RF)	Oral SCC cases and cancer-free controls	53 Cases, 91 controls	Mean: 38.5±7	Not specified/ Not specified	1.Age<br>2.Gender<br>3.Tobacco<br>4.Alcohol	Inadequate	Oral SCC	3 years (1999-2001)	• Smoking MJ was not associated with increased risk of oral SCC (OR=0.3, 95% CI 0.1–1.8) after adjusting for tobacco and alcohol consumption.	High	NHSE-LRO, R and D, and RFP
Rosenblatt 2004[37] Case-control †	Oral SCC cases and controls	407 Cases, 615 controls	18-65	Not specified/ 20% cases, 16% controls	1.Age<br>2.Gender<br>3.Education<br>4.Tobacco<br>5.Alcohol	Adequate	Oral SCC	10 years (1985-1995)	• Smoking MJ was not associated with increased risk of oral SCC with ever used of MJ (OR=0.9, 95% CI 0.6–1.3), total years of use, average frequency of use, years since first use of MJ, or years since last use of MJ after adjusting for key confounders and baseline variables.	Moderate	None
Zhang 1999[36] Case-control †	HNSCC cases and cancer-free controls	173 Cases, 176 controls	Mean: 55.1± 10.4	Not specified/ 17.1 % (370/2159) cases, 45.5% (1358/2985) controls	1.Age<br>2.Gender<br>3.Race<br>4.Education<br>5.Tobacco<br>6.Alcohol	Adequate	HNSCC	2years (1992-1994)	• Smoking MJ was associated with increased risk of HNSCC (OR=2.6, 95% CI 1.1– 6.6) after adjusting for key confounders and baseline variables.	Moderate	NIEHS, NCI or NIDA, NIH, DHHS, UCLJCCF, WF

Flight Attendants Medical Research Institute (FAMRI), International Cancer Research (ICR), Damon Runyon Cancer Research Foundation Clinical Investigator (DRCRFCI), The State of MarylanCigarette Restitution Fund (SMCRF), The National Institute of Dental and Craniofacial Research (NIDCR), The New Zealand Ministry of Health (NMH), The Hawke's Bay Medical Research Foundation (HBMRF), The Alper Research Program for Environmental Genomics of the UCLA Jonsson Comprehensive Cancer Center (ARPEG of the UCLA JCCC), NHS Executive London (NHSE-LRO), Responsive Funding Programme (RFP), Research and Development (R and D), National Institute of Environmental Health Services (NIEHS), National Cancer Institute (NCI or NIDA), National Institutes of Health (NIH), Department of Health and Human Services (DHHS), University of California at Los Angeles Jonsson Cancer Center Foundation (UCLJCCF), The Weissman Fund (WF), Head and Neck Squamous Cell Carcinoma (HNSCC), Nasopharyngeal Cancer (NPC), Marijuana (MJ), Socioeconomic Status (SES), Family History (FH), Oral Squamous Cell Carcinoma (Oral SCC)

*****All comparisons are to never users unless specified otherwise

†We extracted adjusted risk ratio for these studies to use in the meta-analysis

‡All studies used structured questionnaire to assess MJ exposure

© 2019 Ghasemiesfe M et al. *JAMA Network Open*.

eTable 3. Studies that examined exposure to marijuana and development of urogenital cancer

Study Year Design	Study Population	Sample Size	Age (years)	Average MJ exposure/ % MJ only users	Confounders and baseline variables	Adjustment	Outcome Examined	Follow-up	Study Results	Risk of Bias	Funding source
Thomas 2015[41] Prospective	Cohort members from CMHS	34,000 Cases, 48,050 Controls	Mean: 58	Not specified/ 14% (11,491/82,050)	1.Age<br>2.Race<br>3.Ethnicity<br>4.BMI	Inadequate	Bladder cancer	Median: 8.9 years	• Among MJ users, 0.3% (89 cases) developed bladder cancer compared to 0.4% (190 men) of non-smokers (P < .001).<br>• Smoking MJ among MJ-only users was associated with a 45% reduction in bladder cancer (HR=0.55, 95% CI 0.31-1.00, p=0.048) after adjusting for baseline variables.	Moderate	None
Lacson 2012[42] Case-control †	TGCT cases and controls	163 Cases, 292 controls	Mean: 26.5± 3.6	Not specified, Not specified	1.Age<br>2.Race<br>3.Ethnicity<br>4.Education<br>5. Hx of cryptorchidism<br>6.Cocaine<br>7.Amyl nitrite	Adequate	TGCT	7 years (1987-1994)	• Ever use of smoked MJ was associated with increased risk of TGCT (OR=1.94, 95% CI 1.02–3.68) after adjusting for key confounders and baseline variables.<br>• Current MJ smoking was not associated with increased risk of TGCT (OR=1.38, 95% CI 0.67–2.87) while it was associated with past MJ use (OR=2.28, 95% CI 1.17–4.43) after adjusting for key confounders and baseline variables.<br>• Smoking MJ for <10 years was associated with increased risk of TGCT (OR=2.09, 95% CI 1.09–3.98), while it was not associated with ≥10 years of MJ use (OR=1.51, 95%CI: 0.66–3.47) after adjusting for key confounders and baseline variables.	Moderate	NCI
Trabert 2011[43] Case-control †	TGCT cases and controls	187 Cases, 148 controls	Median: 33.5	Not specified/ Not specified	1.Age<br>2.Race<br>3.Hx of cryptorchidism<br>4.Tobacco<br>5.Alcohol	Adequate	TGCT	6 years (1990-1996)	• Ever use of smoked MJ was not associated with increased risk of TGCT (OR=0.7, 95% CI 0.4–1.1) after adjusting for key confounders and baseline variables.<br>• Smoking MJ for < or > ten years was not associated with increased risk of TGCT [(OR=0.6, 95% CI 0.3–1.0), (OR=1.2, 95% CI 0.6–2.8)] after adjusting for key confounders and baseline variables.<br>• Smoking MJ was associated with increased risk of TGCT in frequent MJ	Moderate	The University of Texas, NCI

© 2019 Ghasemiesfe M et al. *JAMA Network Open*.

									users (daily ≥ 1 per day) (OR=2.2; 95% CI, 1.0-5.1) after adjusting for key confounders and baseline variables.		
Daling 2009[44] Case-control †	TGCT cases and cancer-free controls	369 Cases, 979 controls	Range: 18-44	Not specified/ Not specified	1.Age 2.Hx of cryptorchidism 3.Tobacco 4.Alcohol	Adequate	TGCT	7 years (1999-2006)	• Ever use of smoked MJ was associated with increased risk of TGCT (OR=1.3, 95% CI 1.0–1.8) after adjusting for key confounders and baseline variables. • Current MJ smoking was associated with increased risk of TGCT (OR=1.7, 95% CI 1.1–2.5) while it was not associated with past MJ use (OR=1.2, 95% CI 0.9–1.7) after adjusting for key confounders and baseline variables. • Smoking MJ for < or > ten years was associated with increased risk of TGCT [(OR=1.8, 95% CI 1.0–3.3), (OR=1.6, 95% CI 1.1–2.5)] after adjusting for key confounders and baseline variables.	Moderate	NIH, FHCRC
Chacko 2006[45] Case-control	TCC cases and cancer-free controls	52 Cases, 104 controls	Mean: 51.5	48.0± 69.7 joint-years/ 11.6% (6/52)	1.Age 2.Agent orange 3.Radiation 4.Dye	Adequate	TCC	N/A	• Smoking MJ among MJ-only smokers was associated with increased risk of TCC (OR=3.3) after multivariate adjustment for key confounders. • Ever use of smoked MJ was associated with increased risk of TCC (OR=3.4) after multivariate adjustment for key confounders. • Smoking MJ remained statistically significantly associated with TCC (P trend 0.01) by increasing joint-years of MJ use after multivariate adjusting for key confounders.	Low	GCC
Sidney 1997[26] Retrospective §	Participants from KPMCP-NC	64,855	Mean: 33	Not specified/ 40.1% (10,710/26,733)	1.Age 2.Race 3.Education 4.Tobacco 5.Alcohol	Adequate	1.Prostate cancer 2.Cervical cancer	Mean: 8.6 years	• Ever use of smoked MJ among MJ-only smokers was associated with increased risk of prostate cancer (RR=3.1, 95% CI 1.0-9.5) and with a nearly significant increased risk of cervical cancer (RR=1.4, 95% CI 1.0-2.1) compared to non-MJ and non-tobacco smokers after adjusting for alcohol and baseline variables. • Ever use of smoked MJ among MJ-only smokers was associated with a non-significant increased risk of invasive cervical cancer (RR=2.4, 95% CI 0.8-6.7) after adjusting for alcohol and baseline	Moderate	NIDA, NCI, ABMRF

© 2019 Ghasemiesfe M et al. *JAMA Network Open*.

									variables.<br>• Current MJ smoking was associated with increased risk of prostate cancer (RR=4.7, 95% CI 1.4-15.5) and a nearly significant increased risk of cervical cancer (RR=1.6, 95% CI 1.0-2.5).		
Maden 1993[46] Case-control	Penile cancer cases and cancer-free controls	110 Cases, 355 controls	<50 -≥65	Not specified/ Not specified	1.Age<br>2.Tobacco<br>3.Alcohol<br>4.# sexual partners	Adequate	1.Penile cancer	11 years (1979-1990)	• Ever use of smoked MJ was not associated with increased risk of penile cancer (OR=1.5, 95% CI 0.7-3.2) after adjusting for key confounders and baseline variables.<br>• Smoking > 50 times MJ was not associated with increased risk of penile cancer (OR=1, 95% CI 0.3-3.6) after adjusting for alcohol consumption and number of sexual partner.	Moderate	NCI, NIH

National Cancer Institute (NCI), The Fred Hutchinson Cancer Research Center (FHCRC), The Georgia Cancer Coalition (GCC), The US National Institute on Drug Abuse (NIDA), The Alcoholic Beverage Medical Research Foundation (ABMRF), Transitional Cell Carcinoma (TCC), Marijuana (MJ), Testicular Germ Cell Tumor (TGCT), California Men's Health Study (CMHS), Kaiser Permanente Medical Care Program of Northern California (KPMCP-NC), Body Mass Index (BMI), National Institutes of Health (NIH)
*****All comparisons are to never users unless specified otherwise
†We extracted adjusted risk ratio for these studies to use in the meta-analysis
‡All studies used structured questionnaire to assess MJ exposure
§ Not specified route of exposure

© 2019 Ghasemiesfe M et al. *JAMA Network Open*.

eTable 4. Studies that examined exposure to marijuana and development of other cancers

Study Year Design	Study Population	Sample Size	Age (years)	Average MJ exposure/ % of MJ only users	Confounders and baseline variables	Adjustment	Outcome Examined	Follow-up	Study Results	Risk of Bias	Funding source
Chao[47], 2009, Prospective †	Men participants with HIV-1/HHV-8 infection from MACS	1335	33.8	Not specified/ Not specified	1.Age<br>2.Education<br>3.Tobacco<br>4.Alcohol<br>5.# Male sexual partners<br>6.# Lifetime sexual partners<br>7.Anal intercourse<br>8.Condom<br>9.Antiretroviral therapy<br>10.CD4 cell count<br>11.STD	Adequate	KS	(1984-1985), (1987-1991), (2001-2003)	• Recent (last 6 months) and prior (last 5 years) smoking of MJ was not associated with increased risk of KS [(HR=1.0, 95% CI 0.79–1.28), (HR=1.25, 95% CI 0.87-1.79)] after adjusting for key confounders and baseline variables.<br>• Smoking MJ ≥weekly was associated with increased risk of KS (HR=1.52, 95% CI (0.99–2.32) in the 5 years lagged analysis after adjusting for key confounders and baseline variables.<br>• Frequent smoking MJ 5 years prior was not associated with increased risk of KS (HR=1.33 (0.94–1.89).	Moderate	NIDA
Efrid[48], 2004, Prospective	Participants from KPMCP-NC	133,811	Mean: 62.2± 13.5	Not specified/ Not specified	1.Gender<br>2.Race<br>3.Education<br>4.Pipes<br>5.Tobacco<br>6.Alcohol<br>7.Coffee	Inadequate	MPAG	Mean: 13.2± 6.7	• Smoking MJ was associated with increased risk of MPAG among individuals who smoked MJ ≥once a month, (RR=2.8, 95% CI 1.3–6.2, p=0.01) after adjusting for key confounders and baseline variables.<br>• Smoking MJ was associated with increased risk of MPAG among individuals who smoked MJ weekly (RR = 3.2, 95% CI = 1.1–9.2) and monthly (RR = 3.6, 95% CI = 1.3–10.2).	Moderate	NCI
Holly[49], 1999, Case-control	Participants from NCCC	1,281 Cases, 2,095 controls	Median: 58.3	Not specified/ Not specified	1.Age<br>2.Gender<br>3.County of residence	Inadequate	NHL	7 years (1988-1995)	• Smoking MJ was not associated with increased risk of NHL among individuals who smoked MJ ≥ 1000 times, [(Men: OR=0.49, 95% CI 0.31-0.78), (Women: OR=0.71, 95% CI 0.34-1.5)] after adjusting for age.<br>• Smoking MJ was not associated with increased risk of NHL among individuals who smoked MJ ≥ 40 times (OR=0.57, 95% CI 0.44-0.74) after adjusting for age, education, and sex.	High	NCI, NIH
Sidney[26], 1997,	Participants from KPMCP-	64,855	Mean: 33	Not specified/	1.Age<br>2.Race	Adequate	Colorectal cancer	Mean: 8.6 years	• Ever use of smoked MJ among MJ-only smokers was not associated with	Moderate	NIDA, NCI,

© 2019 Ghasemiesfe M et al. *JAMA Network Open.*

Retrospective †	NC			40.1% (10,710/26,733)	3.Education 4.Tobacco 5.Alcohol		Melanoma Breast cancer		increased risk of colorectal cancer [(men: RR=0.7, 95% CI 0.2-2.1), (women: RR=0.3, 95% CI 0.0-2.5)] compared to non-MJ and non-tobacco smokers after adjusting for alcohol and baseline variables. • Ever use of smoked MJ among MJ-only smokers was not associated with increased risk of melanoma [(men: RR=0.5, 95% CI 0.2-1.3), (women: RR=1.0, 95% CI 0.4-2.3)] compared to non-MJ and non-tobacco smokers after adjusting for alcohol and baseline variables. • Ever use of smoked MJ among MJ-only smokers was not associated with increased risk of breast cancer (RR=0.8, 95% CI 0.5-1.3) compared to non-MJ and non-tobacco smokers after adjusting for alcohol and baseline variables.		ABMRF

National Cancer Institute (NCI), The National Institute on Drug Abuse (NIDA), The Alcoholic Beverage Medical Research Foundation (ABMRF), National Institutes of Health (NIH), Kaposi's Sarcoma (KS), Non-Hodgkin's Lymphoma (NHL), Malignant Primary Adult-onset Glioma (MPAG), Multicenter AIDS Cohort Study (MACS), Northern California Cancer center (NCCC), Sexually Transmitted Disease (STD), Kaiser Permanente Medical Care Program of Northern California (KPMCP-NC), Marijuana (MJ)

*****All comparisons are to never users unless specified otherwise

†Not specified route of exposure

‡All studies used structured questionnaire to assess MJ exposure

© 2019 Ghasemiesfe M et al. *JAMA Network Open*.

eAppendix 4. QUALITY ASSESSMENT CRITERIA AND RISK OF BIAS ASSESSMENT

Observational studies: criteria based on the Newcastle-Ottawa scale

Representativeness of the exposed cohort
1 = truly representative of the average patient in the community
1 = somewhat representative of the average patient in the community
0 = selected group of users (e.g., nurses, volunteers)
0 = no description of the derivation of the cohort

Selection of the non-exposed cohort Enter 0 or 1:
1 = drawn from the same community as the exposed cohort
0 = drawn from a different source
0 = no description of the derivation of the non-exposed cohort

Ascertainment of exposure Enter 0 or 1:
1 = biological test (e.g., blood/urine)
1 = structured interview
1 = written self-report that characterizes dose (current or cumulative)
0 = written self-report without quantification of exposure
0 = no description

Precision of Exposure Dose Ascertainment
1 = amount and time
0 = no information about amount and time

Ascertainment of exposure done prospectively or retrospectively
1 = Prospectively
0 = Retrospectively

Demonstration that outcome of interest was not present at start of study, or baseline assessment
1= yes
0 = no

Adjustment for confounding (rendering comparability of cohorts on the basis of the design or analysis)
1 = study accounts/controls for some confounders
2 = complete adjustment for confounders and all relevant baseline characteristics.
0 = no adjustment for potential confounders

Assessment of outcome Enter 0 or 1:
1 = objective measure
1 = validated self-report measures
0 = no information or non-validated measures

Was follow-up long enough for outcomes to occur?
1 = yes (need to define adequate follow-up period for outcome of interest)
0 = no

Adequacy of follow-up of cohorts Enter 0 or 1:
1 = complete follow-up; all subjects accounted for.
1 = subjects lost to follow-up unlikely to introduce bias; small number (less than 20 %) lost, or description was provided of those lost.
0 = follow-up rate < 80% and no description of those lost.
0 = no statement

© 2019 Ghasemiesfe M et al. *JAMA Network Open*.

Case Control Studies: Observational studies: criteria based on the Newcastle-Ottawa scale

Selection

1) Is the case definition adequate?
a) yes, with independent validation
b) yes, e.g. record linkage or based on self-reports
c) no description

2) Representativeness of the cases
a) consecutive or obviously representative series of cases
b) potential for selection biases or not stated

3) Selection of Controls
a) community controls
b) hospital controls
c) no description

4) Definition of Controls
a) no history of disease (endpoint)
b) no description of source

Comparability

1) Comparability of cases and controls on the basis of the design or analysis
a) study controls for tobacco
b) study controls for any additional factors (socioeconomic and socio-demographic factors, relevant baseline factors for outcome of interest)

Exposure

1) Ascertainment of exposure
a) secure record (e.g. surgical records)
b) structured interview where blind to case/control status
c) interview not blinded to case/control status
d) written self-report or medical record only
e) no description

2) Same method of ascertainment for cases and controls
a) yes
b) no

3) Non-Response rate
a) same rate for both groups
b) non-respondents described
c) rate different and no designation

© 2019 Ghasemiesfe M et al. *JAMA Network Open*.

Clinical Trials: Criteria based on the Cochrane risk of bias tool

Domain	Support for judgement
Random sequence generation	Describe the method used to generate the allocation sequence in sufficient detail to allow an assessment of whether it should produce comparable groups.
Allocation concealment	Describe the method used to conceal the allocation sequence in sufficient detail to determine whether intervention allocations could have been foreseen in advance of, or during, enrolment.
Blinding of participants and personnel. Assessments should be made for each main outcome (or class of outcomes).	Describe all measures used, if any, to blind study participants and personnel from knowledge of which intervention a participant received. Provide any information relating to whether the intended blinding was effective.
Blinding of outcome assessment. Assessments should be made for each main outcome (or class of outcomes).	Describe all measures used, if any, to blind outcome assessors from knowledge of which intervention a participant received. Provide any information relating to whether the intended blinding was effective.
Incomplete outcome data. Assessments should be made for each main outcome (or class of outcomes).	Describe the completeness of outcome data for each main outcome, including attrition and exclusions from the analysis. State whether attrition and exclusions were reported, the numbers in each intervention group (compared with total randomized participants), reasons for attrition/exclusions where reported, and any re-inclusions in analyses performed by the review authors.
Selective reporting	State how the possibility of selective outcome reporting was examined by the review authors, and what was found.
Other sources of bias	State any important concerns about bias not addressed in the other domains in the tool.

© 2019 Ghasemiesfe M et al. *JAMA Network Open*.

eTable 5. Risk of Bias Assessment in Cohort and Cross-sectional Studies

Criterion	Chao et al, 2009 (47) (prospective)	Callaghan et al, 2013 (25) (prospective)	Thomas et al, 2015 (41) (prospective)
Representative-ness of the exposed cohort	1 – Participants from an ongoing longitudinal cohort study (Multicenter AIDS Cohort Study (MACS))	1 – Participants from Swedish male conscripts born and military service, the Swedish Patient Register, the National Cause-of-Death Register, the Swedish Total Population Register	1 – Participants recruited from ongoing cohort of the California Men' s Health Study (CMHS)
Selection of the nonexposed Cohort	1 – Unexposed selected from same cohort	1 – Unexposed selected from same cohort	1 – Unexposed selected from same cohort
Ascertainment of Exposure	1 – Structured questionnaire used to ascertain exposure	0 – self-report without adequate quantification	1 – Structured questionnaire used to ascertain exposure
Precision of Exposure Dose Ascertainment	1 – Conducted sampling in 3 stages. Every 6 months, the men in the MACS filled a questionnaire on frequency of substance use: no use (0, reference), monthly or less frequent use (12), and weekly or more frequent use (52) and also complete a physical examination.	1 – Participants filled a questionnaire on ever versus never use of marijuana in lifetime, and lifetime frequency of marijuana use. Users were categorized based on lifetime marijuana use as: never (reference group), once, 2–4 times, 5–10 times, 11–50 times, and more than 50 times (a category defined as ''heavy'' use).	1 – Participants filled a questionnaire on the number of times of cannabis use (none, 1 or 2 times, 3-10 times, 11-99 times, 100-499 times, or >500 times). Cannabis users were characterized as non-use or any use.
Ascertainment of exposure done prospectively or retrospectively	1-Prospectively assessed	1 – Prospectively assessed	1-Prospectively assessed
Demonstration that outcome of interest was not present at start of study, or baseline assessment	1 – Malignancy (Kaposi's Sarcoma (KS) in HIV- and HHV-8-coinfected homosexual men) outcomes are continuously monitored in MACS.	1 – Lung cancer or mortality from lung cancer were the outcomes of interest.	1 – Participants were excluded if they had a history of bladder cancer which was either self-reported or obtained from Kaiser Permanente Surveillance.
Adjustment for Confounding	1 – Adjusted for age, college education, study center, alcohol use, tobacco smoking, number of male sexual partners since the last study visit, lifetime number of sexual partners, receptive anal intercourse and condom use, antiretroviral therapy, CD4 cell count, and sexually transmitted infection score.	1 – Adjusted for tobacco use, alcohol use, respiratory conditions, and socioeconomic status.	0 – Adjusted for age, race, ethnicity, and BMI. Result was reported on cannabis-only smokers.
Assessment of outcome	1- KS was identified by morphology code 9140.3.	1 – Lung cancer outcomes were identified with ICD-8/9/10, ICD-8/9, 162.x, ICD-10, C33.x or C34.x codes	1 – Cancer case ascertainment is expected to be highly valid as the Kaiser Permanente cancer registries fulfill the reporting requirements for the State of California Cancer Registry and the National Cancer Institute SEER program.

© 2019 Ghasemiesfe M et al. *JAMA Network Open*.

Was follow-up long enough for outcomes to occur?	1 – Follow up period of 18 years	1 – Follow up period of 40 years	1 – Follow up period of 8.9 years
Adequacy of follow-up of cohorts	1-Adequate f/u	1-Adequate f/u	1-Adequate f/u
Comments on study quality	**Moderate ROB –** There was inadequate description of quantification of marijuana use and inadequate description of analysis. There was adequate adjustment for key confounders however results were not reported on marijuana-only users.	**High ROB –** There was adequate adjustment for key confounders. Large sample of users but very small sample of marijuana-only users. Lifetime exposure assessment, with results reported based on level of exposure, but exposure levels were minimal. Results were not reported on marijuana-only users and there was one-time assessment of marijuana use, with cancer assessment 40 years later.	**Moderate ROB –** There was adequate assessment of marijuana exposure. Results were classified based on different level of exposure and also reported on cannabis-only smokers. However, there was inadequate adjustment for key confounders (e.g., occupational exposure, medications like pioglitazone) and one-time assessment of marijuana exposure.

© 2019 Ghasemiesfe M et al. *JAMA Network Open*.

eTable 5. Risk of Bias Assessment in Cohort and Cross-sectional Studies *(continued)*

**Criterion**	**Efrid et al, 2004 (48) (prospective)**	**Sidney et al, 1997 (26) (retrospective)**	**Han et al, 2010 (32) (cross-sectional)**
Representative-ness of the exposed cohort	1 – Participants were volunteers from Kaiser Permanente Medical Care Program of Northern California (KPMCP-NC)	1 – Participants were volunteers from Kaiser Permanente Medical Care Program members (KPMCP)	1 – Participants from National Surveys on Drug Use and Health (NSDUH) (2005–2007)
Selection of the nonexposed Cohort	1 – Unexposed selected from same cohort	1 – Unexposed selected from same cohort	1 – Unexposed selected from same cohort
Ascertainment of Exposure	1 – Structured questionnaire used to ascertain exposure	1 – Structured questionnaire used to ascertain exposure	1 – Structured questionnaire used to ascertain exposure
Precision of Exposure Dose Ascertainment	1 – Participants filled a questionnaire on ever versus never use of marijuana. Users were categorized based on the frequency of marijuana use as: never (reference group) and ever users (less than once a month or at least once a month)	1 – Participants filled a questionnaire on if they were current marijuana smokers (smoking currently and more than six times ever), former marijuana smokers (denial of current smoking but admission to having smoked more than six times ever), or nonsmokers (never smoking)	1 – Duration of use of any illicit drugs was measured from the earliest age at initiation to the latest age at last use of any illicit drug (never used, < 1 year, 2–10 years, or 11 years or more).
Ascertainment of exposure done prospectively or retrospectively	1-Prospectively assessed	0 – Retrospectively assessed	0 – Retrospectively assessed
Demonstration that outcome of interest was not present at start of study, or baseline assessment	1 – Participants had no prior history of benign or malignant brain tumors (International Classification of Diseases, 9th revision (ICD-9) [32]: 191.X, 192.1, 194.3, 194.4, 225.2, 227.3, 227.4, 237.0, 237.1, 237.5, 237.6).	1 – Participants excluded if they had cancer subsequent to or within one year prior to the date of HIV/AIDS diagnosis.	0 – N/A
Adjustment for Confounding	0 – Adjusted for cigars, pipes, sex, race, alcohol, education, and coffee, as of baseline questionnaire.	0 – Adjusted for age, race, education, alcohol use, and tobacco cigarette smoking.	1 – Adjusted for durations of non-medical use of pain relievers, tranquilizers, stimulants, and sedatives, duration of alcohol use, duration of tobacco use, daily cigarette smoking history, and other potential confounding factors (age, gender, race/ ethnicity, education, health insurance status, and family income).
Assessment of outcome	1- Primary malignant glioma were identified by International Classification of Diseases for Oncology (ICD-O): 938X/3-948X/3).	1- Incident cancers were determined from computerized databases of confirmed cancer cases maintained by the Northern California Cancer Center and from the Kaiser Permanente Northern California Regional Cancer Registry. Cancer cases were categorized according to ICD-9 codes.	0 – No description

© 2019 Ghasemiesfe M et al. *JAMA Network Open*.

Was follow-up long enough for outcomes to occur?	1 – Follow up period of 13.2 years	1 – Follow up period of 8.6 years	N/A<br>cross-sectional
Adequacy of follow-up of cohorts	1-Adequate f/u	1-Adequate f/u	N/A<br>cross-sectional
Comments on study quality	**Moderate ROB –** Marijuana use was not quantified and there was no description on data collection of marijuana use. There was inadequate adjustment for key confounders (e.g. history of radiation exposure, history of cancer, family history of cancer). Results were not reported on marijuana-only users.	**Moderate ROB –** Marijuana use was not quantified. There was inadequate adjustment for key confounders (e.g. family history of cancer, HPV and other virus infection, genetic syndromes). There was low level of marijuana exposure with limited years of follow up.	**High ROB –** Lung cancer diagnosis was self-reported. Unclear assessment of outcome results. It was also unclear if outcome of interest was present at baseline assessment. Marijuana use was quantified. Results were not reported on marijuana-only users. Inadequate adjustment for key confounders (e.g., occupational exposure).

© 2019 Ghasemiesfe M et al. *JAMA Network Open*.

eTable 6. Risk of Bias in Case-Control Studies

Criteria	Liang et al, 2009 (34)	Aldington et al, 2008 (lung) (27)	Zhang et al, 2015 (29)
Is the case definition adequate?	1 – Defined based on pathological examination	1 – Defined based on clinical-only diagnoses and histopathological examination	1 – Defined based on histological examination
Representativeness of the cases	1 – Cases were patients with primary incident lung cancer (no more than 6 months before the time of patient contact)	1 – Cases were patients with lung cancer (no lung metastasis from a distant primary, or a histological diagnosis of carcinoid or melanoma)	1 – Cases were patients with primary incident lung cancer, pooled from 6 studies
Selection of Controls	1 – Community controls	1 – Community controls	0 – Unclear
Definition of Controls	0 – Inadequate description to confirm that controls had no history of disease	1 – Adequate description to confirm that controls had no history of outcome of interest	0 – Inadequate description to confirm that controls had no history of disease
Comparability of cases and controls based on the design or analysis	1 – Matched to cases on age (± 3 years), gender, and town of residence. Results were adjusted for age and gender, covariates such as race, education, HPV16 serology, family history of cancer, smoking pack-years, and average alcohol drinks per week.	1 – Unclear comparability of cases and controls based on the design/analysis (matched for ± 5 years age and district health boards). Results were adjusted for age, pack-years of cigarette smoking, sex, ethnicity, family history of lung cancer.	1 – Adjusted within studies for age, sex, race, education, and tobacco smoking (never vs. ever) and pack-years of tobacco.
Ascertainment of exposure	1 – Used questionnaire (self-reported) to ascertain exposure	1 – Used questionnaire (self-reported) to ascertain exposure	1 – Used questionnaire (self-reported) to ascertain exposure
Same method of ascertainment for cases and controls	1 – Same method used	1 – Same method used	1 – Same method used
Non-Response rate	0 – No description	0 – No description	0 – No description
Comment:	**Moderate ROB –** Results were not reported on marijuana-only smokers and ascertainment of exposure limited by recall bias. Marijuana use was quantified. Results were reported based on different level of exposure.	**Moderate ROB –** Non-biased selection of cases and controls. Adequate marijuana exposure. Results were not reported on marijuana-only smokers. Marijuana use was quantified. Results were reported based on different level of exposure. No information on exact duration of use in case and control subjects. Ascertainment of exposure limited by recall bias.	**High ROB –** Unclear details of individual studies. Inadequate information on pooling. Limited number of marijuana-only users. Inadequate description of control selection and inadequate description to confirm that controls had no history of disease. No adjustment for occupation, family history of cancer. Inclusion/exclusion criteria unclear. Ascertainment of exposure limited by recall bias.

© 2019 Ghasemiesfe M et al. *JAMA Network Open*.

eTable 6. Risk of Bias in Case-Control Studies *(continued)*

Criterion	Berthiller et al, 2008 (30)	Voiron et al, 2006 (31)	Hashibe et al, 2006 (28)
Is the case definition adequate?	1 – Defined based on histologic, cytologic or radiologic examination	1 – Defined based on histologic or cytology or radiologic examination	1 – Defined based on histologic or radiologic examination
Representativeness of the cases	1 – Cases were patients with hospital enrolled primary incident lung cancer	1 – Cases were patients with hospital enrolled primary incident lung cancer	1 – Cases were patients with new incident lung cancer or upper aerodigestive tract cancer
Selection of Controls	1 – Hospital controls	1 – Hospital controls	1 – Community controls
Definition of Controls	1 – Adequate description to confirm that controls had no history of outcome of interest	1 – Adequate description to confirm that controls had no history of outcome of interest	1 – Adequate description to confirm that controls had no history of outcome of interest
Comparability of cases and controls based on the design or analysis	1 – Matched to cases on case on age, sex, and place of residence and results were adjusted for country, age, tobacco smoking, and occupational exposure.	1 – Adjusted for age, tobacco smoking, and occupational Exposure.	1 – Matched to cases on age decade, gender, and residential neighborhood and results were adjusted for age, gender, race/ethnicity, education, drink-years, tobacco use (ever/never), and pack-years.
Ascertainment of exposure	1 – Used questionnaire (self-reported) to ascertain exposure	1 – Used questionnaire (self-reported) to ascertain exposure	1 – Face-to-face interviews to ascertain exposure
Same method of ascertainment for cases and controls	1 – Same method used	1 – Same method used	1 – Same method used
Non-Response rate	0 – No description	0 – No description	0 – No description
Comment:	**High ROB –** Clear how cases defined, no information on exposure dose and duration collected in one of the pooled case-control studies, missing data were considered as never smokers of cannabis, no definition of ever or former smokers. No adjustment for medical history, family history of cancer. Results were not reported on marijuana-only smokers. Ascertainment of exposure is limited by recall bias.	**High ROB –** None of participants were current marijuana users. No information on exposure dose and duration of use in case and control subjects. Inadequate information on measures of marijuana exposure. No adjustment for sociodemographic factors, diet, environmental factors, medical history, and family history of cancer. Results were not reported on marijuana-only smokers. Ascertainment of exposure limited by recall bias.	**Moderate ROB –** Results were not reported on marijuana-only smokers and ascertainment of exposure limited by recall bias. Marijuana use was quantified. Results were reported based on different level of exposure.

© 2019 Ghasemiesfe M et al. *JAMA Network Open*.

eTable 6. Risk of Bias in Case-Control Studies *(continued)*

Criteria	Lacson et al, 2012 (42)	Trabert et al, 2011 (43)	Feng et al, 2009 (40)	Maden et al, 1993 (46)
Is the case definition adequate?	1 – Defined based on histological examination	1 – Defined based on histological examination	1 – Identified by clinicians in the oncology and radiotherapy departments	1 – Defined based on histological examination
Representativeness of the cases	1 – Cases were men with diagnosed testicular germ cell tumor (TGCT)	1 – Cases were men with incident primary TGCT	1 – Cases were patients with nasopharyngeal cancer (NPC)	1 – Cases were men with diagnosed Penile cancer
Selection of Controls	1 – Community controls	1 – Community controls	1 – Community and hospital controls	1 – Community controls
Definition of Controls	0 – Inadequate description to confirm that controls had no history of outcome of interest	0 – Inadequate description to confirm that controls had no history of outcome of interest	1 – Adequate description to confirm that controls had no history of outcome of interest	1 – Adequate description to confirm that controls had no history of outcome of interest
Comparability of cases and controls based on the design or analysis	1 – Matched to cases on date of birth (within 3 years), race, ethnicity, and neighborhood of residence at the time of diagnosis and results were adjusted for education, religiosity, history of cryptorchidism, ever use of cocaine, and ever use of amyl nitrite.	0 – Matched to cases on age and race and results were adjusted for age, race, prior cryptorchidism, cigarette smoking and alcohol intake.	1 – Matched to cases on center, age, sex, and childhood household type (urban/rural). Analyses were stratified by sex and center and adjusted for age, SES measures, associated dietary factors, and cigarettes smoked per day.	1 – Matched to cases on age and date of diagnosis and results were adjusted for age, alcohol consumption, cigarette smoking (never, former, or current), and number of sexual partners.
Ascertainment of exposure	1 – Used questionnaire (self-reported) to ascertain exposure	1 – Used questionnaire (self-reported) to ascertain exposure	1 – Used questionnaire (self-reported) to ascertain exposure	1 – Used questionnaire (self-reported) to ascertain exposure
Same method of ascertainment for cases and controls	1 – Same method used	1 – Same method used	1 – Same method used	1 – Same method used
Non-Response rate	0 – No description	0 – No description	1 – 10% (primary reason was old age)	1 – 44.7% of cases and 29.7% of controls
Comment:	**Moderate ROB –** Inadequate measurement of total marijuana exposure. Results were not reported on marijuana-only smokers. Good selection of cases and	**Moderate ROB –** Marijuana use was not quantified. Inadequate measurement for total marijuana exposure. Results were not reported on marijuana-only smokers. Ascertainment of	**Moderate ROB –** Results were not reported on marijuana-only smokers. The selection of cases and controls had potential bias. Inconsistent adjustment for important confounders. No	**Moderate ROB –** Inadequate measurement for total marijuana exposure. Results were not reported on marijuana-only smokers. Ascertainment of

© 2019 Ghasemiesfe M et al. *JAMA Network Open*.

	controls and good ascertainment of baseline data. Ascertainment of exposure limited by recall bias.	exposure limited by recall bias.	adjustment for recreational drug use, occupational exposure, or alcohol. Ascertainment of exposure limited by recall bias.	exposure limited by recall bias.

© 2019 Ghasemiesfe M et al. *JAMA Network Open*.

eTable 6. Risk of Bias in Case-Control Studies *(continued)*

Criterion	Daling et al, 2009 (44)	Aldington et al, 2008 (H&N) (35)	Llewellyn et al, 2004-(AA) (39)
Is the case definition adequate?	1 – Defined based on Oncology, topography and histology examination	1 – Identified from hospital databases and the New Zealand Cancer Registry	1 – Identified by accessing the Thames Cancer Registry (TCR) database (with pathological confirmation)
Representativeness of the cases	1 – Cases were patients with invasive testicular germ cell tumor (TGCT)	1 – Cases were patients with confirmed prevalent or incident head and neck cancer (no metastasis from a distant primary other than head and neck or a histologic diagnosis of carcinoid, melanoma, or adenocystic carcinomas)	1 – Cases were young patients with diagnosed squamous cell carcinoma (no salivary glands, nasopharynx and hypopharynx cancer)
Selection of Controls	1 – Community controls	1 – Community controls	1 – Community controls
Definition of Controls	1 – Adequate description to confirm that controls had no history of outcome of interest	1 – Adequate description to confirm that controls had no history of outcome of interest	1 – Adequate description to confirm that controls had no history of outcome of interest
Comparability of cases and controls based on the design or analysis	1 – Matched to cases in five-year age groups, and within the same three counties. Results were adjusted for age, reference year, alcohol use, current smoking, and history of cryptorchidism.	1 – Matched to cases in five-year age groups to the expected national incidence of head and neck cancer and district health boards to increase the study efficiency and results were adjusted for age, sex, ethnicity alcohol consumption, income, and pack years of cigarette smoking.	1 – Matched to cases on sex, area of residence and age (within 2 year). Adjustments made for tobacco and alcohol consumption.
Ascertainment of exposure	1 – Used questionnaire (self-reported) to ascertain exposure	1 – Used questionnaire (self-reported) to ascertain exposure	1 – Used questionnaire (self-reported) to ascertain exposure
Same method of ascertainment for cases and controls	1 – Same method used	1 – Same method used	1 – Same method used
Non-Response rate	0 – 32.5% in cases and 47.8% in controls	0 – 24% in cases and 34% in controls	0 – No description (any patients found to be deceased or those who had moved overseas were excluded)
Comment:	**Moderate ROB –** No information on exposure dose and inadequate quantification of lifelong marijuana exposure. Results were not reported on marijuana-only smokers. No definition of ever, current or former smokers. Ascertainment of exposure limited by recall bias.	**Moderate ROB –** Results were not reported on marijuana-only smokers. Adequate information on exposure dose and duration of use. Marijuana use was quantified, and results reported on different level of exposure and duration of use. Baseline characteristics and key confounders adjusted for in the analysis. Ascertainment of exposure limited by recall bias.	**High ROB –** No description on quantification of marijuana use. Inadequate information to confirm if cases or controls comparable. No adjustment for sociodemographic factors, diet, environmental factors including exposure to environmental smoke, medical history (selected chronic diseases), and family history of cancer. Results were not reported on marijuana-only

© 2019 Ghasemiesfe M et al. *JAMA Network Open*.

			smokers.

© 2019 Ghasemiesfe M et al. *JAMA Network Open*.

eTable 6. Risk of Bias in Case-Control Studies *(continued)*

Criterion	Gillison et al, 2008 (33)	Chacko et al, 2006 (45)	Llewellyn et al, 2004-(RF) (38)
Is the case definition adequate?	1 – Defined based on histologic examination	0 – No description	1 – Identified by participating consultants in their respective units
Representativeness of the cases	1 – Cases were patients with newly diagnosed HNSCC at the outpatient otolaryngology clinic of the Johns Hopkins Hospital (oral cavity, paranasal sinus, pharynx, or larynx or an unknown primary HNSCC)	1 – Cases were patients with transitional cell carcinoma of the bladder	1 – Cases were young patients with diagnosed SCC of the lip, intra-oral sites and oropharynx/tonsil (no salivary glands, nasopharynx and hypopharynx cancer)
Selection of Controls	1 – Community controls (as an outpatient for any benign condition at the same otolaryngology clinic)	1 – Community controls (population of men aged 60 and younger presenting to the urology clinic for other complaints)	1 – Community controls
Definition of Controls	1 – Adequate description to confirm that controls had no history of outcome of interest	1 – Adequate description to confirm that controls had no history of outcome of interest	1 – Adequate description to confirm that controls had no history of outcome of interest
Comparability of cases and controls based on the design or analysis	1 – Matched to cases on sex, age (5-year intervals) and race. Results were adjusted for race, tobacco use, alcohol use, tooth loss, frequency of tooth brushing, and number of oral sex partners.	1 – Matched to cases on age (date of birth within 12 months). Results adjusted for other potential risk factors, including agent orange exposure, radiation exposure, and dye exposure.	0 – Matched for sex, area of residence and within 2 years of the cases' age and results were adjusted for tobacco and alcohol consumption.
Ascertainment of exposure	1 – Used questionnaire (self-reported) to ascertain exposure	1 – Used questionnaire (self-reported) to ascertain exposure	1 – Used questionnaire (self-reported) to ascertain exposure
Same method of ascertainment for cases and controls	1 – Same method used	1 – Same method used	1 – Same method used
Non-Response rate	0 – No description	0 – No description	1 – 20% of cases
Comment:	**Low ROB** – Adequate information on exposure dose and duration of use. Marijuana use was quantified, and results reported by level of exposure and duration of use. Results were reported on marijuana-only users. Adequate adjustment for baseline characteristics and key. Ascertainment of exposure limited by recall bias.	**Low ROB –** Good selection of cases and controls and good ascertainment of baseline. Results were reported on marijuana-only users. Adequate assessment of marijuana exposure and adjustment for confounders. Results were reported based on different level of exposure. Ascertainment of exposure limited by recall bias.	**High ROB** – No information on exposure dose and duration of use. Results were not reported on marijuana-only users. Inadequate information to confirm if cases or controls comparable. No adjustment for sociodemographic factors, diet, environmental factors, medical history (selected chronic diseases), and family history of cancer. Ascertainment of exposure limited by recall bias.

© 2019 Ghasemiesfe M et al. *JAMA Network Open*.

eTable 6. Risk of Bias in Case-Control Studies *(continued)*

Criteria	Rosenblatt et al, 2004 (37)	Zhang et al, 1999 (36)	Holly et al, 1999 (49)
Is the case definition adequate?	1 – Identified by using data, and biological specimens	1 – Defined based on histologic examination	1 – Identified by using the Northern California Cancer Center's rapid case ascertainment system and confirmed by independent pathology review
Representativeness of the cases	1 – Cases were patients with with first, incident oral squamous cell carcinoma (OSCC)	1 – Cases were patients with untreated first primary squamous cell carcinoma of the head and neck	1 – Cases were patients with non-Hodgkin's lymphoma
Selection of Controls	1 – Community controls	1 – Community controls	1 – Community controls
Definition of Controls	0 – Inadequate description to confirm that controls had no history of outcome of interest	1 – Adequate description to confirm that controls had no history of outcome of interest	0 – Inadequate description to confirm that controls had no history of outcome of interest
Comparability of cases and controls based on the design or analysis	1 – Matched to cases on age and sex. Results adjusted for sex, education, birth year, average number of alcoholic drinks/week, and pack-years of cigarette smoking.	1 – Matched to cases on age- and sex- and results adjusted for age, gender, race, education, heavy alcohol drinking, pack-years of tobacco cigarette smoking, and passive smoking.	0 – Matched to cases on sex, county of residence, and age within 5 years and results were adjusted for age.
Ascertainment of exposure	1 – Used questionnaire (self-reported) to ascertain exposure	1 – Used questionnaire (self-reported) to ascertain exposure	1 – Face-to-face interviews to ascertain exposure
Same method of ascertainment for cases and controls	1 – Same method used	1 – Same method used	1 – Same method used
Non-Response rate	1 – 40.3% of cases and 38% of controls	0 – No description (failed to report frequency of use, no information on years of use)	0 – 44% of cases
Comment:	**Moderate ROB –** Results were not reported on marijuana-only users. No description of measurement of marijuana exposure. Marijuana use was quantified, and results reported on different level of exposure and duration of use. Baseline characteristics and key confounders adjusted for in their analysis. Ascertainment of exposure limited by recall bias.	**Moderate ROB –** Results were not reported on marijuana-only users. Adequate information on exposure dose and duration of use in case and control subjects. Marijuana use was quantified, and results reported on different level of exposure and duration of use. Baseline characteristics and key confounders adjusted for in their analysis. Ascertainment of exposure limited by recall bias.	**High ROB –** No information on exposure dose and duration of use. There was inadequate marijuana exposure. Inadequate information to confirm if cases or controls comparable. Inadequate adjustment for key confounders. Results were not reported on marijuana-only users.

© 2019 Ghasemiesfe M et al. *JAMA Network Open*.

eFigure 1. Funnel plot: Head and neck squamous cell cancer case-control studies

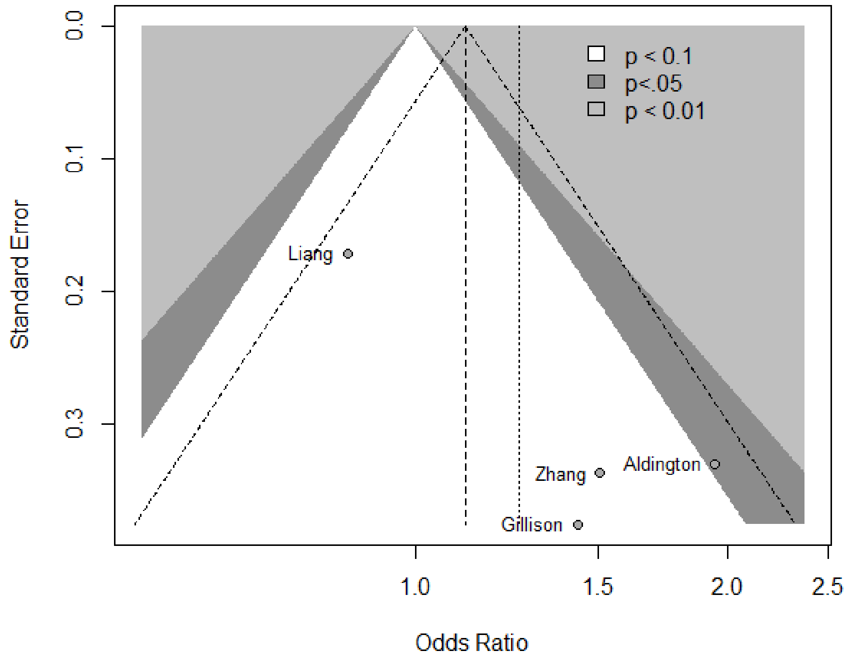

eFigure 2. Funnel plot: Testicular germ cell tumor case-control studies

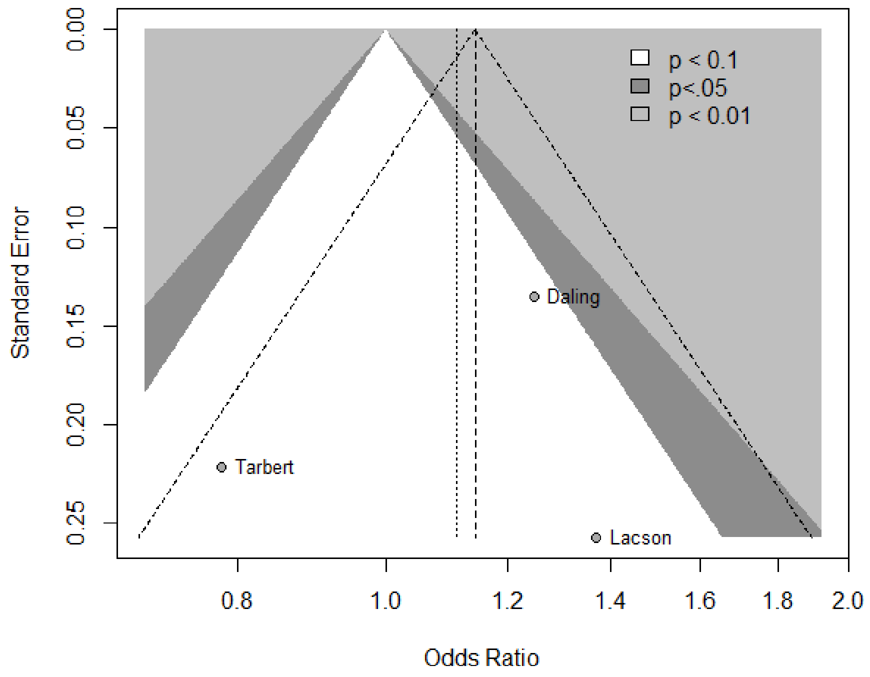

© 2019 Ghasemiesfe M et al. *JAMA Network Open*.

eFigure 3. Funnel plot: Testicular germ cell tumor case-control studies (>10 years use)

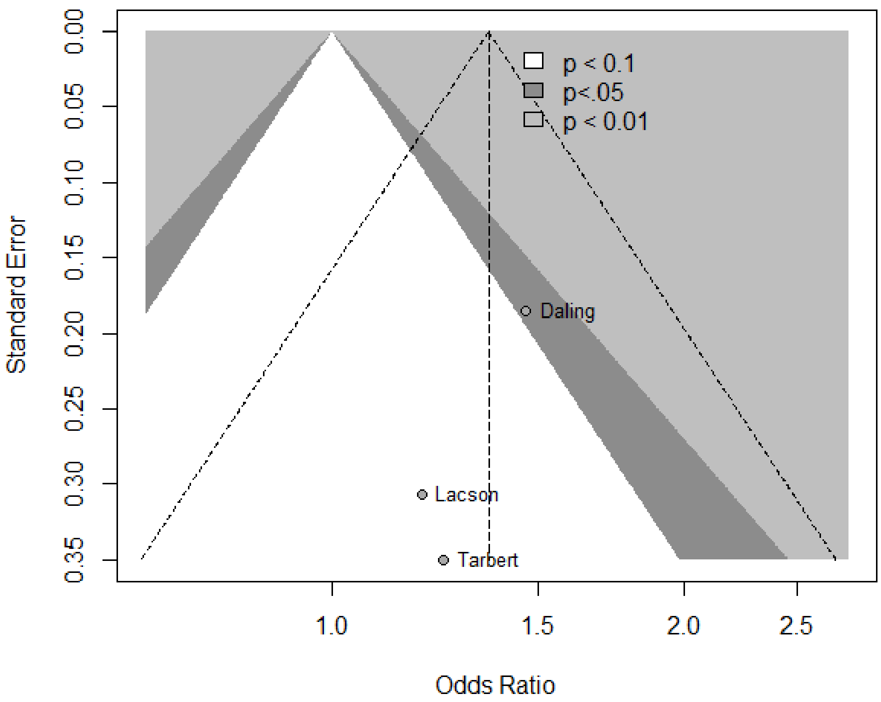

eFigure 4. Funnel plot: Testicular germ cell tumor case-control studies (seminoma)

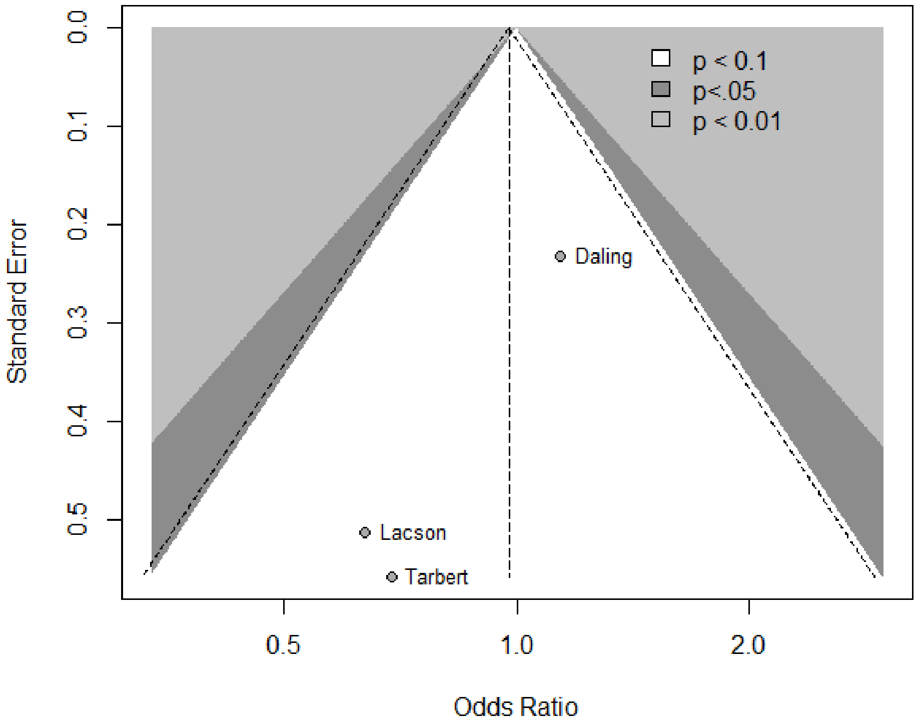

© 2019 Ghasemiesfe M et al. *JAMA Network Open*.

eReferences

1. National Survey on Drug Use and Health. Results from the 2017 national survey on drug use and health: detailed tables. Sep 7, 2018.Accessed at https://www.samhsa.gov/data/sites/default/files/cbhsqreports/NSDUHDetailedTabs2017/NSDUHDetailedTabs2017.pdf.
2. National Institutes of Health. Prevalence of marijuana use among U.S. adults doubles over past decade [press release]. 21 October 2015.
3. Azofeifa A, Mattson ME, Schauer G, et al. National estimates of marijuana use and related indicators—National Survey on Drug Use and Health, United States, 2002-2014. *MMWR Surveill Summ* 2016;65:1-28.[PMID:27584586].doi:10.15585/mmwr.ss6511a1.
4. Steigerwald S, Wong PO, Cohen BE, et al. Smoking, vaping, and use of edibles and other forms of marijuana among US adults. *Ann Intern Med* 2018 Dec18;169(12):890-892.[PMID:30167665].doi:10.7326/M18-1681.
5. Hoffmann D, Brunnemann KD, Gori GB, et al. On the carcinogenicity of marijuana smoke. In: Runeckles VC, ed. *Recent Advances in Phytochemistry. Boston: Springer-Verlag* 1975:63-81.
6. Moir D, Rickert WS, Levasseur G, et al. A comparison of mainstream and sidestream marijuana and tobacco cigarette smoke produced under two machine smoking conditions. *Chem Res Toxicol* 2007;*21*(2): 494-502.[PMID:18062674].
7. Wu TC, Tashkin DP, Djahed B, et al. Pulmonary hazards of smoking marijuana as compared with tobacco. *N Engl J Med* 1988;*318*(6): 347-351.[PMID:3340105].
8. Tashkin DP, Gliederer F, Rose J, et al. Tar, CO and Δ9-THC delivery from the 1st and 2nd halves of a marijuana cigarette. *Pharmacol Biochem Behav* 1991;*40*(3):657-661.[PMID:1666924].
9. Tashkin DP, Baldwin GC, Sarafian T, et al. Respiratory and immunologic consequences of marijuana smoking. *J. Clin. Pharmacol* 2002;*42*(S1): 71S-81S.[PMID:12412839].
10. Heron MP. Deaths: Leading causes for 2016. 2018. Accessed at https://stacks.cdc.gov/view/cdc/57988.
11. Lortet-Tieulent J, Sauer AG, Siegel RL, et al. State-level cancer mortality attributable to cigarette smoking in the United States. *JAMA Intern Med* 2016;*176*(12): 1792-1798.[PMID:27775761].doi:10.1001/jamainternmed.2016.6530.
12. Barsky SH, Roth MD, Kleerup EC, et al. Histopathologic and molecular alterations in bronchial epithelium in habitual smokers of marijuana, cocaine, and/or tobacco. *J Natl Cancer Inst* 1998;90:1198-205.[PMID:9719080].
13. Śledziński P, Zeyland J, Słomski R, et al. The current state and future perspectives of cannabinoids in cancer biology. *Cancer Med* 2018;*7*(3): 765-775.[PMID:29473338].doi:10.1002/cam4.1312.
14. Pellati F, Borgonetti V, Brighenti V, et al. Cannabis sativa L. and nonpsychoactive cannabinoids: their chemistry and role against oxidative stress, inflammation, and cancer. *BioMed Res. Int* 2018 Dec4:1691428.[PMID:30627539].doi: 10.1155/2018/1691428.
15. Moher D, Liberati A, Tetzlaff J, et al. PRISMA Group. Preferred reporting items for systematic reviews and meta-analyses: the PRISMA statement. *Ann Intern Med* 2009;151:264-9.[PMID:19622511].
16. Blachly PH. Effects of decriminalization of marijuana in Oregon. *Ann N Y Acad Sci* 1976;282:405-15.[PMID:1071391].
17. IMS Institute for Healthcare Informatics. Global Use of Medicines: Outlook through 2017. 2017. http://www.quotidianosanita.it/allegati/allegato1501906.pdf.
18. Bramer WM, Giustini D, de Jonge GB, et al. De-duplication of database search results for systematic reviews in EndNote. *J Med Libr Assoc* 2016;104:240-3.10.3163/1536-5050.104.3.014
19. Wells GA, Shea B, O'Connell D, et al. The Newcastle-Ottawa Scale (NOS) for assessing the quality of nonrandomized studies in meta-analyses. The Ottawa Hospital Research Institute. 2014.Accessed at www.ohri.ca/programs/clinical_epidemiology/oxford.asp on 29 September 2017.
20. Paule RC, Mandel J. Consensus values and weighting factors. *J Res Natl Bur Stand* 1982;87:377-85.

© 2019 Ghasemiesfe M et al. *JAMA Network Open.*

21. Hartung J, Knapp G. A refined method for the meta-analysis of controlled clinical trials with binary outcome. *Stat Med* 2001;20: 3875-89.[PMID:11782040].
22. Higgins JP, Thompson SG, Deeks JJ, et al. Measuring inconsistency in meta-analyses. *BMJ* 2003;327:557-60.[PMID:12958120].
22. Harbord RM, Egger M, Sterne JA. A modified test for small-study effects in meta-analyses of controlled trials with binary endpoints. *Stat Med* 2006; 25(20):3443-57.
24. Berkman ND, Lohr KN, Ansari M, et al. Grading the Strength of a Body of Evidence When Assessing Health Care Interventions for the Effective Health Care Program of the Agency for Healthcare Research and Quality: An Update. Methods Guide for Comparative Effectiveness Reviews. (Prepared by the RTI-UNC Evidence-based Practice Center under contract no. 290-2007-10056-I.) AHRQ publication no. 13(14)-EHC130-EF. Rockville: Agency for Healthcare Research and Quality; November 2013.Accessed at https://ahrq-ehc-application.s3.amazonaws.com/media/pdf/methods-guidance-grading-evidence_methods.pdf on 12 October 2017.
25. Callaghan RC, Allebeck P, Sidorchuk A. Marijuana use and risk of lung cancer: a 40-year cohort study. *Cancer Causes Control* 2013 Oct;24(10):1811-20.[PMID:23846283].doi:10.1007/s10552-013-0259-0.
26. Sidney S, Quesenberry CP, Friedman GD, et al. Marijuana use and cancer incidence (California, United States). *Cancer Causes Control* 1997; *8*(5): 722-728.[PMID:9328194].
27. Aldington S, Harwood M, Cox B, et al. Cannabis use and risk of lung cancer: a case–control study. *Eur Respir J* 2008; *31*(2): 280-286.[PMID:18238947].doi:10.1183/09031936.00065707.
28. Hashibe M, Morgenstern H, Cui Y, et al. Marijuana use and the risk of lung and upper aerodigestive tract cancers: results of a population-based case-control study. *Cancer* Epidemiol *Biomarkers* Prev 2006; *15*(10): 1829-1834.[PMID:17035389].
29. Zhang LR, Morgenstern H, Greenland S, et al. Cannabis and Respiratory Disease Research Group of New Zealand and Brhane, Y. Cannabis smoking and lung cancer risk: Pooled analysis in the International Lung Cancer Consortium. *Int. J. Cancer* 2015; *136*(4): 894-903. [PMID:24947688].doi:10.1002/ijc.29036.
30. Berthiller J, Straif K, Boniol M, et al. Cannabis smoking and risk of lung cancer in men: a pooled analysis of three studies in Maghreb*. J. Thorac. Oncol* 2008 Dec;3(12):1398-403.[PMID:19057263].doi:10.1097/JTO.0b013e31818ddcde.
31. Voirin N, Berthiller J, Benhaïm-Luzon V, et al. Risk of lung cancer and past use of cannabis in Tunisia. *J. Thorac. Oncol* 2006;*1*(6): 577-579.[PMID:17409920].
32. Han B, Gfroerer JC, Colliver JD. Associations between duration of illicit drug use and health conditions: results from the 2005–2007 national surveys on drug use and health. *Ann Epidemiol* 2010;*20*(4): 289-297.[PMID: 20171900].doi: 10.1016/j.annepidem.2010.01.003.
33. Gillison ML, D'souza G, Westra W, et al. Distinct risk factor profiles for human papillomavirus type 16–positive and human papillomavirus type 16–negative head and neck cancers. *J. Natl. Cancer Inst* 2008; *100*(6): 407-420.[PMID:18334711].doi: 10.1093/jnci/djn025.
34. Liang C, McClean MD, Marsit C, et al. A population-based case-control study of marijuana use and head and neck squamous cell carcinoma. *Cancer Prev Res* 2009;*2*(8): 759-768.[PMID:19638490].doi: 10.1158/1940-6207.CAPR-09-0048.
35. Aldington S, Harwood M, Cox B, et al. Cannabis use and cancer of the head and neck: case-control study. *Otolaryngol Head Neck Surg* 2008;*138*(3): 374-380.[PMID:18312888].doi: 10.1016/j.otohns.2007.12.002.
36. Zhang ZF, Morgenstern H, Spitz MR, et al. Marijuana use and increased risk of squamous cell carcinoma of the head and neck. *Cancer Epidemiol Biomarkers Prev* 1999;*8*(12): 1071-1078.[PMID:10613339].
37. Rosenblatt KA, Daling JR, Chen C, et al. Marijuana use and risk of oral squamous cell carcinoma. *Cancer Res* 2004;*64*(11): 4049-4054.[PMID:15173020].
38. Llewellyn CD, Johnson NW, Warnakulasuriya KAAS. Risk factors for oral cancer in newly diagnosed patients aged 45 years and younger: a case–control study in Southern England. *J Oral Pathol Med* 2004;*33*(9): 525-532.[PMID:15357672].

© 2019 Ghasemiesfe M et al. *JAMA Network Open*.

39. Llewellyn CD, Linklater K, Bell J, et al. An analysis of risk factors for oral cancer in young people: a case-control study. *Oral Oncol* 2004;*40*(3): 304-313.[PMID:14747062].
40. Feng BJ, Khyatti M, Ben-Ayoub W, et al. Cannabis, tobacco and domestic fumes intake are associated with nasopharyngeal carcinoma in North Africa. *Br. J. Cancer* 2009;*101*(7): 1207.[PMID:19724280].doi:10.1038/sj.bjc.6605281.
41. Thomas AA, Wallner LP, Quinn VP, et al. Association between cannabis use and the risk of bladder cancer: results from the California Men's Health Study. *J Urol* 2015;*85*(2): 388-393. [PMID:25623697]. doi:10.1016/j.urology.2014.08.060.
42. Lacson JCA, Carroll JD, Tuazon E, et al. Population-based case-control study of recreational drug use and testis cancer risk confirms an association between marijuana use and nonseminoma risk. *Cancer* 2012;*118*(21): 5374-5383.[PMID:22965656].doi:10.1002/cncr.27554.
43. Trabert B, Sigurdson AJ, Sweeney AM, et al. Marijuana use and testicular germ cell tumors. *Cancer* 2011;*117*(4): 848-853.[PMID:20925043].doi:10.1002/cncr.25499.
44. Daling JR, Doody DR, Sun X, et al. Association of marijuana use and the incidence of testicular germ cell tumors. *Cancer* 2009;*115*(6): 1215-1223.[PMID:19204904].doi:10.1002/cncr.24159.
45. Chacko JA, Heiner JG, Siu W, et al. Association between marijuana use and transitional cell carcinoma. *J Urol* 2006;*67*(1): 100-104.[PMID:16413342].
46. Maden C, Sherman KJ, Beckmann AM, et al. History of circumcision, medical conditions, and sexual activity and risk of penile cancer. *J. Natl. Cancer Inst* 1993;*85*(1): 19-24.[PMID:8380060].
47. Chao C, Jacobson LP, Jenkins FJ, et al. Recreational drug use and risk of Kaposi's sarcoma in HIV-and HHV-8-coinfected homosexual men. *AIDS Res Hum Retroviruses* 2009;*25*(2): 149-156. [PMID:19108691]. doi:10.1089/aid.2008.0196.
48. Efird JT, Friedman GD, Sidney S, et al. The risk for malignant primary adult-onset glioma in a large, multiethnic, managed-care cohort: cigarette smoking and other lifestyle behaviors. *J. Neuro-Oncol* 2004;*68*(1): 57-69.[PMID:15174522].
49. Holly EA, Lele C, Bracci PM, et al. Case-control study of non-Hodgkin's lymphoma among women and heterosexual men in the San Francisco Bay Area, California. *Am J Epidemiol* 1999;*150*(4): 375-389.[PMID:10453814].
50. Mehra R, Moore BA, Crothers K, et al. The association between marijuana smoking and lung cancer: a systematic review. *Arch. Intern. Med* 2006;*166*(13): 1359-1367.[PMID:16832000].
51. Martinasek MP, McGrogan JB, Maysonet A. A systematic review of the respiratory effects of inhalational marijuana. *Respir Care* 2016;*61*(11): 1543-1551.[PMID:27507173].
52. De Carvalho MFF, Dourado MR, Fernandes IB, et al. Head and neck cancer among marijuana users: A meta-analysis of matched case–control studies. *Arch. Oral Biol* 2015;*60*(12): 1750-1755. [PMID:26433192]. doi:10.1016/j.archoralbio.2015.09.009.
53. Gurney J, Shaw C, Stanley J, et al. Cannabis exposure and risk of testicular cancer: a systematic review and meta-analysis. *BMC cancer* 2015;*15*(1): 897. [PMID:26560314]. doi:10.1186/s12885-015-1905-6.
54. Gandhi S, Vasisth G, Kapoor A. Systematic review of the potential role of cannabinoids as antiproliferative agents for urological cancers. *Can Urol Assoc J* 2017;*11*(3-4): E138. [PMID:28515817]. doi:10.5489/cuaj.4371.
55. Rajanahally S, Raheem O, Rogers M, et al. The relationship between cannabis and male infertility, sexual health, and neoplasm: a systematic review. *Andrology* 2019.
56. Sarafian TA, Magallanes JAM, Shau H, et al. Oxidative stress produced by marijuana smoke: an adverse effect enhanced by cannabinoids. *Am J Respir* Cell *Mol Biol* 1999;*20*(6): 1286-1293.[PMID:10340948].
57. Roth MD, Arora A, Barsky SH, et al. Airway inflammation in young marijuana and tobacco smokers. *Am J Respir Crit Care Med* 1998;157:928-37.[PMID:9517614].
58. Fligiel SE, Roth MD, Kleerup EC, et al. Tracheobronchial histopathology in habitual smokers of cocaine, marijuana, and/or tobacco. *Chest* 1997;112:319-26.[PMID:9266864].
59. Kaufman DW, Palmer JR, Rosenberg L, et al. Tar content of cigarettes in relation to lung cancer. *Am J Epidemiol* 1989;*129*(4):703-711.[PMID:2923118].

© 2019 Ghasemiesfe M et al. *JAMA Network Open*.

60. Yoshie Y, Ohshima H. Synergistic induction of DNA strand breakage by cigarette tar and nitric oxide. *Carcinogenesis* 1997;*18*(7): 1359-1363. [PMID:9230280].
61. Zang EA, Wynder EL. Cumulative tar exposure. A new index for estimating lung cancer risk among cigarette smokers. *Cancer* 1992;*70*(1): 69-76.[PMID:1606549].
62. Mattson ME, Pollack ES, Cullen JW. What are the odds that smoking will kill you?. *Am J Public Health* 1987;*77*(4): 425-431.[PMID:3826460].
63. Abuhasira R, Shbiro L, Landschaft Y. Medical use of cannabis and cannabinoids containing products–Regulations in Europe and North America. *Eur. J. Intern. Med* 2018; Mar1;49:2-6. [PMID:29329891]. doi:10.1016/j.ejim.2018.01.001.
64. Trivers KF, Phillips E, Gentzke AS, et al. Prevalence of cannabis use in electronic cigarettes among US youth. *JAMA Pediatr* 2018;*172*(11): 1097-1099.[PMID:30242366].
65. Borodovsky JT, Lee DC, Crosier BS, et al. US cannabis legalization and use of vaping and edible products among youth. *Drug and alcohol dependence* 2017;*177*:299-306. [PMID:28662974]. doi:10.1016/j.drugalcdep.2017.02.017.
66. Budney AJ, Sargent JD, Lee DC. Vaping cannabis (marijuana): parallel concerns to e-cigs?. *Addiction* 2015;*110*(11): 1699-1704.[PMID:26264448].doi:10.1111/add.13036.
67. Eng CH, Yu K, Lucas J, et al. Ammonia derived from glutaminolysis is a diffusible regulator of autophagy. *Sci. Signal* 2010;*3*(119): .ra31-ra31. [PMID:20424262]. doi:10.1126/scisignal.2000911.
68. Bloor RN, Wang TS, Španěl P, et al. Ammonia release from heated 'street'cannabis leaf and its potential toxic effects on cannabis users. *Addiction* 2008;*103*(10):1671-1677. [PMID:18705690]. doi:10.1111/j.1360-0443.2008.02281.x.
69. Spinelli JB, Yoon H, Ringel AE, et al. Metabolic recycling of ammonia via glutamate dehydrogenase supports breast cancer biomass. *Science* 2017;*358*(6365):941-946. [PMID:29025995]. doi:10.1126/science.aam9305.
70. Felberbaum, M. FDA warns companies marketing unproven products, derived from marijuana, that claim to treat or cure cancer. November 01, 2017. Accessed at https://www.fda.gov/newsevents/newsroom/pressannouncements/ucm583295.htm.
71. Shi S, Brant AR, Sabolch A, et al. False News of a Cannabis Cancer Cure. *Cureus* 2019; *11*(1). [PMID:30931189]. doi:10.7759/cureus.3918.

© 2019 Ghasemiesfe M et al. *JAMA Network Open*.

eAppendix 5. List of excluded studies

1. Babalonis, S., Haney, M., Malcolm, R.J., Lofwall, M.R., Votaw, V.R., Sparenborg, S. and Walsh, S.L., 2017. Oral cannabidiol does not produce a signal for abuse liability in frequent marijuana smokers. *Drug and alcohol dependence*, *172*, pp.9-13.
2. Pergam, S.A., Woodfield, M.C., Lee, C.M., Cheng, G.S., Baker, K.K., Marquis, S.R. and Fann, J.R., 2017. Cannabis use among patients at a comprehensive cancer center in a state with legalized medicinal and recreational use. *Cancer*, *123*(22), pp.4488-4497.
3. Imtiaz, S., Shield, K.D., Roerecke, M., Cheng, J., Popova, S., Kurdyak, P., Fischer, B. and Rehm, J., 2016. The burden of disease attributable to cannabis use in Canada in 2012. *Addiction*, *111*(4), pp.653-662.
4. Fischer, B., Imtiaz, S., Rudzinski, K. and Rehm, J., 2015. Crude estimates of cannabis-attributable mortality and morbidity in Canada–implications for public health focused intervention priorities. *Journal of public health*, *38*(1), pp.183-188.
5. Smith, S., Janitz, A. and Campbell, J., 2016. Epidemiology of testicular cancer in oklahoma and the United States. *The Journal of the Oklahoma State Medical Association*, *109*(7-8), p.385.
6. Bhattacharyya, S., Mandal, S., Banerjee, S., Mandal, G.K., Bhowmick, A.K. and Murmu, N., 2015. Cannabis smoke can be a major risk factor for early-age laryngeal cancer—a molecular signaling-based approach. *Tumor Biology*, *36*(8), pp.6029-6036.
7. De Carvalho, M.F.F., Dourado, M.R., Fernandes, I.B., Araújo, C.T.P., Mesquita, A.T. and Ramos-Jorge, M.L., 2015. Head and neck cancer among marijuana users: A meta-analysis of matched case–control studies. *Archives of Oral Biology*, *60*(12), pp.1750-1755.
8. Hall, W., 2015. What has research over the past two decades revealed about the adverse health effects of recreational cannabis use?. *Addiction*, *110*(1), pp.19-35.
9. Penson, D.F., 2015. Re: Association between Cannabis Use and the Risk of Bladder Cancer: Results from the California Men's Health Study. *The Journal of urology*.
10. Marks, M.A., Chaturvedi, A.K., Kelsey, K., Straif, K., Berthiller, J., Schwartz, S.M., Smith, E., Wyss, A., Brennan, P., Olshan, A.F. and Wei, Q., 2014. Association of marijuana smoking with oropharyngeal and oral tongue cancers: pooled analysis from the INHANCE consortium. *Cancer Epidemiology and Prevention Biomarkers*, *23*(1), pp.160-171.
11. Lacson, J.C.A., Carroll, J.D., Tuazon, E., Castelao, E.J., Bernstein, L. and Cortessis, V.K., 2012. Population-based case-control study of recreational drug use and testis cancer risk confirms an association between marijuana use and nonseminoma risk. *Cancer*, *118*(21), pp.5374-5383.
12. Hazekamp, A. and Heerdink, E.R., 2013. The prevalence and incidence of medicinal cannabis on prescription in The Netherlands. *European journal of clinical pharmacology*, *69*(8), pp.1575-1580.
13. Marcus, D.M., Jani, A.B. and Rossi, P.J., 2013. Population-based case-control study of recreational drug use and testis cancer risk confirms an association between marijuana use and nonseminoma risk. *Cancer*, *119*(6), pp.1284-1284.
14. Andreotti, G., Liu, E., Gao, Y.T., Safaeian, M., Rashid, A., Shen, M.C., Wang, B.S., Deng, J., Han, T.Q., Zhang, B.H. and Hsing, A.W., 2011. Medical history and the risk of biliary tract cancers in Shanghai, China: implications for a role of inflammation. *Cancer Causes & Control*, *22*(9), p.1289.
15. Chang, G., Meadows, M.E., Jones, J.A., Antin, J.H. and Orav, E.J., 2010. Substance use and survival after treatment for chronic myelogenous leukemia (CML) or myelodysplastic syndrome (MDS). *The American journal of drug and alcohol abuse*, *36*(1), pp.1-6.
16. Berthiller, J., Lee, Y.C.A., Boffetta, P., Wei, Q., Sturgis, E.M., Greenland, S., Morgenstern, H., Zhang, Z.F., Lazarus, P., Muscat, J. and Chen, C., 2009. Marijuana smoking and the risk of head and neck cancer: pooled analysis in the INHANCE consortium. *Cancer Epidemiology and Prevention Biomarkers*, *18*(5), pp.1544-1551.
17. Thompson, A.L., Gerhardt, C.A., Miller, K.S., Vannatta, K. and Noll, R.B., 2009. Survivors of childhood cancer and comparison peers: The influence of peer factors on later externalizing behavior in emerging adulthood. *Journal of Pediatric Psychology*, *34*(10), pp.1119-1128.
18. Dahlstrom, K.R., Little, J.A., Zafereo, M.E., Lung, M., Wei, Q. and Sturgis, E.M., 2008. Squamous cell carcinoma of the head and neck in never smoker–never drinkers: a descriptive epidemiologic study. *Head & Neck: Journal for the Sciences and Specialties of the Head and Neck*, *30*(1), pp.75-84.
19. Brook, J.S., Stimmel, M.A., Zhang, C. and Brook, D.W., 2008. The association between earlier marijuana use and subsequent academic achievement and health problems: A longitudinal study. *The American Journal on Addictions*, *17*(2), pp.155-160.
20. Ahrens, A.G. and Bressi, T., 2007. Marijuana as promoter for oral cancer? More than a suspect. *Addictive Disorders & Their Treatment*, *6*(3), pp.117-119.

© 2019 Ghasemiesfe M et al. *JAMA Network Open*.

21. Bluhm, E.C., Daniels, J., Pollock, B.H. and Olshan, A.F., 2006. Maternal use of recreational drugs and neuroblastoma in offspring: a report from the Children's Oncology Group (United States). *Cancer Causes & Control*, *17*(5), pp.663-669.
22. Roth, M.D., Arora, A., Barsky, S.H., Kleerup, E.C., Simmons, M. and Tashkin, D.P., 1998. Airway inflammation in young marijuana and tobacco smokers. *American journal of respiratory and critical care medicine*, *157*(3), pp.928-937.
23. Tanimowo, M.O. and Onaolapo, Y.A., 2007. The pattern of tobacco use among non-pulmonary tuberculosis patients attending a chest clinic in South-Western Nigeria. *Nigerian journal of clinical practice*, *10*(4), pp.314-318.
24. Chen, A.L., Chen, T.J., Braverman, E.R., Acuri, V., Kerner, M., Varshavskiy, M., Braverman, D., Downs, W.B., Blum, S.H., Cassel, K. and Blum, K., 2008. Hypothesizing that marijuana smokers are at a significantly lower risk of carcinogenicity relative to tobacco-non-marijuana smokers: evidenced based on statistical reevaluation of current literature. *Journal of psychoactive drugs*, *40*(3), pp.263-272.
25. Trivers, K.F., Mertens, A.C., Ross, J.A., Steinbuch, M., Olshan, A.F. and Robison, L.L., 2006. Parental marijuana use and risk of childhood acute myeloid leukaemia: a report from the Children's Cancer Group (United States and Canada). *Paediatric and perinatal epidemiology*, *20*(2), pp.110-118.
26. Lee, P.N. and Forey, B.A., 2003. Why are lung cancer rate trends so different in the United States and United kingdom?. *Inhalation toxicology*, *15*(9), pp.909-949.
27. Hashibe, M., Ford, D.E. and Zhang, Z.F., 2002. Marijuana smoking and head and neck cancer. *The Journal of Clinical Pharmacology*, *42*(S1), pp.103S-107S.
28. Khalsa, J.H., Genser, S., Francis, H. and Martin, B., 2002. Clinical consequences of marijuana. *The Journal of Clinical Pharmacology*, *42*(S1), pp.7S-10S.
29. Jemal, A., Chu, K.C. and Tarone, R.E., 2001. Recent trends in lung cancer mortality in the United States. *Journal of the National Cancer Institute*, *93*(4), pp.277-283.
30. Johnson, N., 2001. Tobacco use and oral cancer: a global perspective. *Journal of dental education*, *65*(4), pp.328-339.
31. Wen, W.Q., Shu, X.O., Steinbuch, M., Severson, R.K., Reaman, G.H., Buckley, J.D. and Robison, L.L., 2000. Paternal military service and risk for childhood leukemia in offspring. *American journal of epidemiology*, *151*(3), pp.231-240.
32. Zhang, Z.F., Morgenstern, H., Spitz, M.R., Tashkin, D.P., Yu, G.P., Hsu, T.C. and Schantz, S.P., 2000. Environmental tobacco smoking, mutagen sensitivity, and head and neck squamous cell carcinoma. *Cancer Epidemiology and Prevention Biomarkers*, *9*(10), pp.1043-1049.
33. Duarte, J.G., do Nascimento, A.F., Pantoja, J.G. and Chaves, C.P., 1999. Chronic inhaled cocaine abuse may predispose to the development of pancreatic adenocarcinoma. *The American journal of surgery*, *178*(5), pp.426-427.
34. Fung, M., Gallagher, C. and Machtay, M., 1999. Lung and aero-digestive cancers in young marijuana smokers. *Tumori*, *85*(2), pp.140-142.
35. Barsky, S.H., Roth, M.D., Kleerup, E.C., Simmons, M. and Tashkin, D.P., 1998. Histopathologic and molecular alterations in bronchial epithelium in habitual smokers of marijuana, cocaine, and/or tobacco. *JNCI: Journal of the National Cancer Institute*, *90*(16), pp.1198-1205.
36. Ammenheuser, M.M., Berenson, A.B., Babiak, A.E., Singleton, C.R. and Whorton Jr, E.B., 1998. Frequencies of hprt mutant lymphocytes in marijuana-smoking mothers and their newborns. *Mutation Research/Fundamental and Molecular Mechanisms of Mutagenesis*, *403*(1-2), pp.55-64.
37. Fligiel, S.E., Roth, M.D., Kleerup, E.C., Barsky, S.H., Simmons, M.S. and Tashkin, D.P., 1997. Tracheobronchial histopathology in habitual smokers of cocaine, marijuana, and/or tobacco. *Chest*, *112*(2), pp.319-326.
38. Hall, W. and Nelson, J., 1996. Correlates of the perceived health risks of marijuana use among Australian adults. *Drug and Alcohol Review*, *15*(2), pp.137-143.
39. Bhatia, S. and Neglia, J.P., 1995. Epidemiology of childhood acute myelogenous leukemia. *Journal of pediatric hematology/oncology*, *17*(2), pp.94-100.
40. Sridhar, K.S., Raub, W.A., Weatherby, N.L., Metsch, L.R., Surratt, H.L., Inciardi, J.A., Duncan, R.C., Anwyl, R.S. and McCoy, C.B., 1994. Possible role of marijuana smoking as a carcinogen in the development of lung cancer at a young age. *Journal of psychoactive drugs*, *26*(3), pp.285-288.
41. A World Health Organizadon Demonstration Project, 1994. A phase II study of delta-9-tetrahydrocannabinol for appetite stimulation in cancer-associated anorexia. *Journal of palliative care*, *10*(1), pp.14-18.
42. Darling, M.R. and Arendorf, T.M., 1993. Effects of cannabis smoking on oral soft tissues. *Community dentistry and oral epidemiology*, *21*(2), pp.78-81.
43. Grufferman, S., Schwartz, A.G., Ruymann, F.B. and Maurer, H.M., 1993. Parents' use of cocaine and marijuana and increased risk of rhabdomyosarcoma in their children. *Cancer Causes & Control*, *4*(3), pp.217-224.
44. Donald, P.J., 1991. Advanced malignancy in the young marijuana smoker. In *Drugs of abuse, immunity, and immunodeficiency* (pp. 33-46). Springer, Boston, MA.

© 2019 Ghasemiesfe M et al. *JAMA Network Open*.

45. Kuijten, R.R., Bunin, G.R., Nass, C.C. and Meadows, A.T., 1990. Gestational and familial risk factors for childhood astrocytoma: results of a case-control study. *Cancer research*, *50*(9), pp.2608-2612.
46. Robison, L.L., Buckley, J.D., Daigle, A.E., Wells, R., Benjamin, D., Arthur, D.C. and Hammond, G.D., 1989. Maternal drug use and risk of childhood nonlymphoblastic leukemia among offspring. An epidemiologic investigation implicating marijuana (a report from the Childrens Cancer Study Group). *Cancer*, *63*(10), pp.1904-1911.
47. Fligiel, S.E., Venkat, H., Gong Jr, H. and Tashkin, D.P., 1988. Bronchial pathology in chronic marijuana smokers: a light and electron microscopic study. *Journal of psychoactive drugs*, *20*(1), pp.33-42.
48. Gong Jr, H., Fligiel, S., Tashkin, D.P. and Barbers, R.G., 1987. Tracheobronchial changes in habitual, heavy smokers of marijuana with and without tobacco. *American Journal of Respiratory and Critical Care Medicine*, *136*(1), pp.142-149.
49. Donald, P.J., 1986. Marijuana smoking—possible cause of head and neck carcinoma in young patients. *Otolaryngology—Head and Neck Surgery*, *94*(4), pp.517-521.
50. Lozada, F., Silverman Jr, S., Migliorati, C.A., Conant, M.A. and Volberding, P.A., 1983. Oral manifestations of tumor and opportunistic infections in the acquired immunodeficiency syndrome (AIDS): findings in 53 homosexual men with Kaposi's sarcoma. *Oral Surgery, Oral Medicine, Oral Pathology*, *56*(5), pp.491-494.
51. Tennant Jr, F.S., 1979. Histopathologic and clinical abnormalities of the respiratory system in chronic hashish smokers. *Problems Of Drug Dependence*, p.309.
52. Taylor 3rd, F.M., 1988. Marijuana as a potential respiratory tract carcinogen: a retrospective analysis of a community hospital population. *Southern Medical Journal*, *81*(10), p.1213.
53. Schydlower, M. "Breast masses in adolescents." *American family physician* 25.2 (1982): 141-145.
54. Almadori, G., Paludeeri, G., Cerullo, M., Orraviani, F. and D'alatri, L., 1990. Marijuana smoking as a possible cause of tongue carcinoma in young patients. *The Journal of Laryngology & Otology*, *104*(11), pp.896-899.
55. McKallip, R.J., Nagarkatti, M. and Nagarkatti, P.S., 2005. Δ-9-tetrahydrocannabinol enhances breast cancer growth and metastasis by suppression of the antitumor immune response. *The Journal of Immunology*, *174*(6), pp.3281-3289.
56. Behavioral risk factors for head and neck cancers identified. (2008). *J Am Dent Assoc, 139*(5), 540-541.
57. Awengen, D.F., 1993. Marijuana and malignant tumors of the upper aerodigestive tract in young patients. On the risk assessment of marijuana. *Laryngo-rhino-otologie*, *72*(5), p.264.
58. Bigay-Gamé, L., Bota, S., Greillier, L., Monnet, I., Madroszyk, A., Corre, R., Mastroianni, B., Falchero, L., Mazières, J., Colineaux, H. and Lepage, B., 2018. Characteristics of Lung Cancer in Patients Younger than 40 Years: A Prospective Multicenter Analysis in France. *Oncology*, *95*(6), pp.337-343.

© 2019 Ghasemiesfe M et al. *JAMA Network Open*.
